# Global population attributable fraction of potentially modifiable risk factors for mental disorders: a meta-umbrella systematic review

**DOI:** 10.1038/s41380-022-01586-8

**Published:** 2022-04-28

**Authors:** Elena Dragioti, Joaquim Radua, Marco Solmi, Celso Arango, Dominic Oliver, Samuele Cortese, Peter B. Jones, Jae Il Shin, Christoph U. Correll, Paolo Fusar-Poli

**Affiliations:** 1 Pain and Rehabilitation Centre and Department of Health, Medicine and Caring Sciences, Linköping University, Linköping, Sweden; 2 Early Psychosis: Interventions and Clinical-detection (EPIC) Lab, Department of Psychosis Studies, Institute of Psychiatry, Psychology & Neuroscience, King’s College London, London, UK; 3 Imaging of Mood- and Anxiety-Related Disorders (IMARD) Group, Institut d’Investigacions Biomèdiques August Pi i Sunyer, Mental Health Networking Biomedical Research Centre (CIBERSAM), Barcelona, Spain; 4 Department of Clinical Neuroscience, Centre for Psychiatric Research and Education, Karolinska Institutet, Stockholm, Sweden; 5 Department of Psychiatry, University of Ottawa, Ottawa, ON Canada; 6 Department of Mental Health, The Ottawa Hospital, Ottawa, ON Canada; 7 Centre for Innovation in Mental Health, School of Psychology, Faculty of Environmental and Life Sciences, University of Southampton, Southampton, UK; 8 Department of Child and Adolescent Psychiatry, Institute of Psychiatry and Mental Health, Hospital General Universitario Gregorio Marañón, Madrid, Spain; 9 Health Research Institute (IiGSM), School of Medicine, Universidad Complutense de Madrid, Madrid, Spain; 10 Biomedical Research Center for Mental Health (CIBERSAM), Madrid, Spain; 11 Clinical and Experimental Sciences (CNS and Psychiatry), Faculty of Medicine, University of Southampton, Southampton, UK; 12 Solent NHS Trust, Southampton, UK; 13 Hassenfeld Children’s Hospital at NYU Langone, New York, NY USA; 14 Division of Psychiatry and Applied Psychology, School of Medicine, University of Nottingham, Nottingham, UK; 15 Department of Psychiatry, University of Cambridge, Cambridge, UK; 16 CAMEO Early Intervention Service, Cambridgeshire and Peterborough National Health Service Foundation Trust, Cambridge, UK; 17 Department of Pediatrics, Yonsei University College of Medicine, Seoul, South Korea; 18 Department of Pediatrics, Severance Children’s Hospital, Seoul, South Korea; 19 Department of Psychiatry, Zucker Hillside Hospital, Northwell Health, Glen Oaks, NY USA; 20 Department of Psychiatry and Molecular Medicine, Zucker School of Medicine at Hofstra/Northwell, Hempstead, NY USA; 21 Center for Psychiatric Neuroscience, Feinstein Institute for Medical Research, Manhasset, NY USA; 22 Department of Child and Adolescent Psychiatry, Charité Universitätsmedizin, Berlin, Germany; 23 OASIS Service, South London and Maudsley NHS Foundation Trust, London, UK; 24 Department of Brain and Behavioral Sciences, University of Pavia, Pavia, Italy

**Keywords:** Prognostic markers, Psychiatric disorders

## Abstract

Numerous risk factors for mental disorders have been identified. However, we do not know how many disorders we could prevent and to what extent by modifying these risk factors. This study quantifies the Population Attributable Fraction (PAF) of potentially modifiable risk factors for mental disorders. We conducted a PRISMA 2020-compliant (Protocol: https://osf.io/hk2ag) meta-umbrella systematic review (Web of Science/PubMed/Cochrane Central Register of Reviews/Ovid/PsycINFO, until 05/12/2021) of umbrella reviews reporting associations between potentially modifiable risk factors and ICD/DSM mental disorders, restricted to highly convincing (class I) and convincing (class II) evidence from prospective cohorts. The primary outcome was the global meta-analytical PAF, complemented by sensitivity analyses across different settings, the meta-analytical Generalised Impact Fraction (GIF), and study quality assessment (AMSTAR). Seven umbrella reviews (including 295 meta-analyses and 547 associations) identified 28 class I–II risk associations (23 risk factors; AMSTAR: 45.0% high-, 35.0% medium-, 20.0% low quality). The largest global PAFs not confounded by indication were 37.84% (95% CI = 26.77–48.40%) for childhood adversities and schizophrenia spectrum disorders, 24.76% (95% CI = 13.98–36.49%) for tobacco smoking and opioid use disorders, 17.88% (95% CI = not available) for job strain and depression, 14.60% (95% CI = 9.46–20.52%) for insufficient physical activity and Alzheimer’s disease, 13.40% (95% CI = 7.75–20.15%) for childhood sexual abuse and depressive disorders, 12.37% (95% CI = 5.37–25.34%) for clinical high-risk state for psychosis and any non-organic psychotic disorders, 10.00% (95% CI = 5.62–15.95%) for three metabolic factors and depression, 9.73% (95% CI = 4.50–17.30%) for cannabis use and schizophrenia spectrum disorders, and 9.30% (95% CI = 7.36–11.38%) for maternal pre-pregnancy obesity and ADHD. The GIFs confirmed the preventive capacity for these factors. Addressing several potentially modifiable risk factors, particularly childhood adversities, can reduce the global population-level incidence of mental disorders.

## Introduction

A large-scale meta-analysis found that the global onset of the first mental disorder occurs before age 14 in one-third (34.6%), before age 18 in half (48.4%), and before age 25 in almost two-thirds (62.5%) of cases, with a peak onset age of 14.5 years and a median age at onset of 18 years across all mental disorders [1]. Due to the suboptimal efficacy of interventions after the onset of mental disorders [2], primary prevention is particularly promising in young people [3]. It encompasses: (i) targeted strategies in individuals at clinical high risk (indicated interventions) [4–6] or those asymptomatic who have significant risk factors (selective interventions) [7–9], or (ii) public health strategies in the general population (universal interventions) [7].

Primary prevention requires a robust aetiopathological knowledge of the natural history of a disorder [10], but mental disorders are intrinsically complex conditions. Although a genetic predisposition is evident, it explains only a small proportion of the phenotypic variance [11–13]; environmental factors underlie much of the phenotypic variation [14]. Individual studies exploring non-purely genetic risk factors for mental disorders have grown over the past decades, to the point that numerous umbrella reviews (i.e., systematic reviews of meta-analyses [15–18]) have summarised the consistency and magnitude of these risk factors [19–22]. As umbrella reviews can robustly rank the credibility of the evidence [23], controlling at the same time for several biases [19–22], they are considered at the top of the hierarchy to evaluate epidemiological evidence [18, 24]. Despite these potentials, the associations reported by umbrella reviews are not directly informative for preventive interventions. Unmasking the power of preventative approaches [25] requires assessing the proportional reduction in population-level disease (Population Attributable Fraction, PAF) [26–29] that would occur if a given risk factor is eliminated in an ideal exposure scenario [29] (https://www.who.int/healthinfo/global_burden_disease/metrics_paf/en/). The PAF, which is influenced by the prevalence of the exposure (risk factor), estimates the epidemiologic contribution of a risk factor to a certain disease [30], informing the prioritisation of preventive targets across diverse prevalence settings (e.g. in low–middle-income countries or in specific sociodemographic groups) [29]. To our best knowledge, no study has estimated the meta-analytic PAF of the most robust risk factors for mental disorders.

We fill this gap by quantifying the consistency and magnitude of the PAF for the most robust non-purely genetic and potentially modifiable risk factors across all mental disorders. We combined published umbrella reviews ranking robust risk factors for mental disorders with global population-level prevalence data and bespoke meta-analytical methods.

## Methods

### Search strategy and selection criteria

We conducted a PRISMA 2020-compliant [31, 32] (eMethods 1) meta-umbrella systematic review; an umbrella review of umbrella reviews [33] (protocol: https://osf.io/hk2ag). Two researchers (ED, MS) independently searched Web of Science (Clarivate Analytics) databases (including the Web of Science Core Collection/BIOSIS Citation Index/MEDLINE/KCI-Korean Journal Database/SciELO Citation Index/Russian Science Citation Index), PubMed, the Cochrane Central Register of Reviews, and Ovid/PsycINFO databases, from inception to 05/12/2021, using: “umbrella review” and (“risk” OR “protect*”, see eBox1). Records identified were screened based on title and abstract; full texts of the relevant records were assessed for inclusion. The references of records included were additionally screened.

Studies included were: (a) umbrella reviews [16, 17]; (b) reporting quantitative data from prospective cohort studies on the association between non-purely genetic risk factors and (ICD/DSM-any version) mental disorders, based on established criteria for classifying the credibility of the evidence [19–22] (see below).

Studies excluded were: (a) systematic reviews or meta-analyses other than quantitative umbrella reviews, individual studies, clinical cases, conference proceedings, and study protocols; (b) umbrella reviews addressing outcomes other than the onset of an established mental disorder (e.g., those related to clinical outcomes such as relapse, remission or treatment response [34, 35]); (c) umbrella reviews employing other classification approaches, such as GRADE [36], because these umbrellas do not present quantitative results from prospective cohort studies only; (d) umbrella reviews addressing pure genetic factors or biomarkers because genetic/biomarker association is tested with other analytical approaches.

Corresponding authors were contacted to clarify data overlaps. When two papers presented overlapping datasets on the same risk factor for the same disorder, only the paper with the largest dataset was retained.

### Measures and data extraction

Two of us (ED, MS) independently extracted a predetermined set of variables characterising each umbrella review, including first author and publication year, number of meta-analyses included, median number of individual studies and cases (with interquartile range) per association in each meta-analysis included, the overall number of risk factors investigated, and the range of years for which the evidence was reviewed.

Additional variables were extracted to characterise the association between risk factors and mental disorders. Each risk factor was pragmatically defined as originally operationalized by each individual study, without redefining it unless strictly necessary to improve reporting clarity (eTable 1). Since each risk factor (e.g., smoking) can be associated with multiple outcomes (e.g., lung and pancreatic cancer), the total number of risk associations tested in umbrella reviews typically exceeds the number of risk factors [37].

We also recorded the specific mental disorder and matched it with the corresponding ICD-10 diagnostic block (eMethods 2). Furthermore, we recorded the number of individual studies and cases analysed per association and the association’s strength as risk ratios (RRs) ±  95% confidence intervals (CIs). Finally, we extracted the class of evidence (class I or II) [13, 18–20, 38] as reported for each association (see below), but only focused on risk factors (protective factors were reversed) that: (i) could be potentially modifiable as clinically evaluated, (ii) were not affected by survival bias, and (iii) were derived from prospective cohort analyses. The latter criterion was applied to specifically deal with the problem of reverse causation that may affect, for example, case–control studies [21].

### Strategy for data synthesis

We presented the associations stratified across the corresponding ICD-10 diagnostic blocks. The classification of the credibility of the evidence was defined according to established criteria [13, 18–20, 38]: prospective class I, convincing (number of cases >1000, *P* < 10^−6^, *I*^2^ < 50%, 95% prediction interval excluding the null, no small-study effects, and no excess significance bias); prospective class II, highly suggestive (number of cases >1000, *P* < 10^−6^, largest study with a statistically significant effect, and class I criteria not met). We indicated whether there could be confounding by indication, e.g., associations between a medical treatment and a mental disorder could be confounded by an underlying medical condition, which would have increased the indication for medical treatment and the risk of the mental disorder [39]. We recorded the quality of the included meta-analyses using the AMSTAR (A Measurement Tool to Assess Systematic Reviews) tool, as reported in original umbrella reviews [40].

The global PAF analysis (primary outcome) was then conducted [29]. To retrieve robust prevalence data (±95% CIs), we adopted a systematic multistep approach. We preferably used estimates from the Global Burden of Disease Study (GBD, 2019) (http://ghdx.healthdata.org), followed by GBD 2015, a catalogue of global health, causes, demographic data, and vital statistics for both global and county profiles previously established in epidemiological research [41–45]. When GBD prevalences were not available, we favoured global reports of population-level prevalences by international agencies (e.g., World Health Organization, Centers for Disease Control and Prevention, European Centre for Disease Prevention and Control, etc.) [46] (https://data.cdc.gov; https://www.ecdc.europa.eu/en), followed by meta-analyses/systematic reviews, and then individual population-based studies (eTables 2 and 3). Additional computations were also performed to generate prevalence data as detailed in eMethods 3 and eFigures 1–4. All primary analyses followed the pre-specified protocol. Sensitivity analyses additionally tested the impact of variable prevalence in different settings (eMethods 4).

We appraised the quality of individual studies reporting prevalence using a modified version of a critical appraisal tool for systematic reviews addressing prevalence items [47].

The calculation of the PAF was based on Levin’s formula [48], which requires the RR estimate and the prevalence (P) of the risk factor [49].

$$PAF = \frac{{P\left( {RR - 1} \right)}}{{P\left( {RR - 1} \right) + 1}}$$

Even if odds ratios (OR) are very similar to RRs when the incidence of an outcome is low, we preferred converting all ORs to RRs using a standard formula [50]. In all, 95% CIs for the PAFs were derived using a method similar to Daly’s [51]. Specifically, for each risk factor, we created 50,000 random RRs according to the RR 95%CI and 50,000 random prevalences according to the prevalence 95% CI. We then combined the random RRs and prevalences to derive 50,000 PAF estimations, from which we derived the PAF 95% CI.

While the PAF assumes a perfect intervention that eradicates the exposure (i.e. 100% reduction of the prevalence of the risk factors) [30], complete removal of exposure is often unrealistic. Therefore, we performed additional (secondary) analyses by computing the GIF (Generalised Impact Fraction, also called the generalised attributable fraction) for factors with the largest PAFs (as the GIF is ≤PAF, for smaller PAFs, the GIF analysis would be futile) and not confounded by indication. The GIF estimates the proportional reduction in disease incidence given a graded reduction in the prevalence of a risk factor [30, 52].

All analyses were conducted using Stata 17 (StataCorp. 2017 Stata Statistical Software: Release 17. College Station, TX) and R (version 4.0.3).

## Results

### Database

**Fig. 1:**
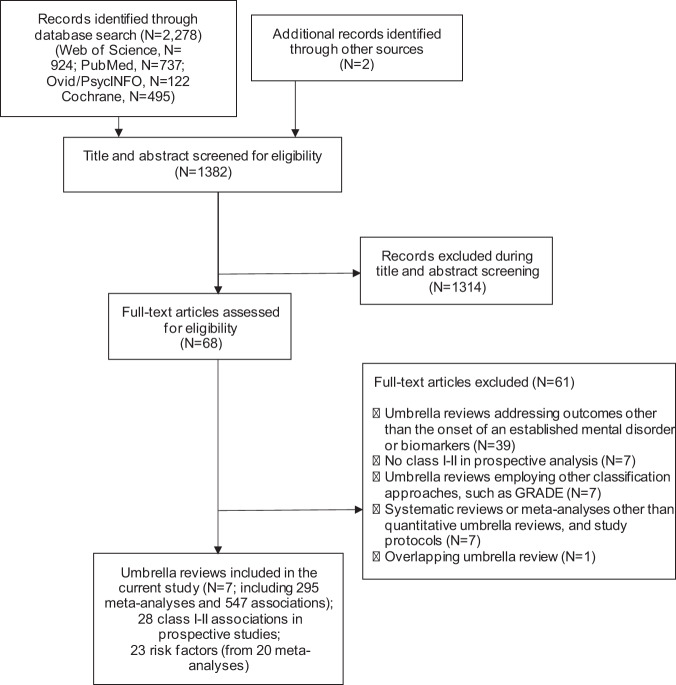
PRISMA flow chart outlining study selection process. The flow chart  maps out the number of records identified, included and excluded, and the reasons for exclusions.

Overall, 2278 records were retrieved, 1382 suitable papers were screened after duplicates were removed, and seven umbrella reviews were finally included after examining 68 for depth eligibility [17, 37, 53–57]. (see Fig. 1 and eTable 4). Included umbrella reviews were published 2017–2021, with individual studies published 1995–2020. The seven eligible umbrella reviews (eTable 5) included 295 meta-analyses (median = 43, interquartile range = 35–55) and 547 associations between putative risk factors and mental disorders that were analysed.

### Characteristics of the included umbrella reviews

Among the 547 associations, 30 were of class I, 40 were of class II, 70 were of class III, 227 of class IV, and 180 were non-significant in the main analysis. However, only 28 risk associations of class I–II (16 class I and 12 class II), relating to 23 risk factors (eTable 2 for definitions) from 20 meta-analyses survived in prospective analysis (after excluding non-modifiable risk factors, such as widowhood, and factors affected by survival biases, such as the history of cancer), and were included in the current study. Table 1 summarises the associations of the 23 risk factors and mental disorders that have been included in the current study, stratified by ICD-10 diagnostic blocks.

**Table 1:** Evidence for associations between non-purely genetic risk and mental disorders restricted to factors with class of evidence I–II in prospective analyses, stratified by ICD-10 diagnostic block.

Risk factor	Mental disorder	Number of individual studies (cases)	Strength of association (RR)	95% CI	Class of evidence in prospective analysis	Quality (AMSTAR)
Organic, including symptomatic, mental disorders						
T2DM	Vascular dementia	14 (1396)	2.28	1.94–2.66	I	High
Depression	Any dementia	23 (2781)	1.86	1.61–2.14	I	High
Depression in elderhood	Any dementia	22 (4782)	1.83	1.65–2.03	I	Medium
Depression in elderhood	Alzheimer’s disease	15 (3348)	1.64	1.40–1.92	I	Medium
Low frequency of social contacts	Any dementia	8 (1122)	1.57	1.32–1.85	I	Medium
T2DM	Alzheimer’s disease	21 (3537)	1.54	1.39–1.72	I	High
Benzodiazepines use*	Any dementia	5 (11,741)	1.49	1.30–1.72	I	High
Depression	Alzheimer’s disease	15 (1461)	1.72	1.39–2.13	II	High
Insufficient physical activity**	Alzheimer’s disease	9 (1358)	1.62	1.38–1.91	II	Medium
T2DM	Any dementia	22 (15,707)	1.60	1.43–1.79	II	High
Mental and behavioural disorders due to psychoactive substance use						
Tobacco smoking	Opioid use disorder	6 (1834)	2.61	1.79–3.79	II	Low
Schizophrenia, schizotypal, and delusional disorders						
CHR-P	Any non-organic psychotic disorder	9 (1226)	9.30	4.91–17.66	I	High
Cannabis use	Schizophrenia spectrum disorders	6 (1294)	3.84	2.34–6.29	II	High
Childhood adversities	Schizophrenia spectrum disorders	8 (4085)	2.57	1.94–3.40	II	Medium
Mood (affective) disorders						
Sexual dysfunction	Depressive disorders	6 (5488)	2.49	NA	I	High
Four or five metabolic risk factors	Depressive disorders	8 (1191)	1.98	1.56–2.53	I	Low
Childhood physical abuse	Depressive disorders	4 (3054)	1.89	1.70–2.09	I	Medium
Job strain	Depressive disorders	7 (1909)	1.73	1.44–2.06	I	Medium
Obesity	Depressive disorders	8 (7673)	1.33	1.20–1.47	I	Low
Childhood sexual abuse	Depressive disorders	7 (3621)	2.31	1.72–3.10	II	Medium
Three metabolic risk factors	Depressive disorders	8 (3014)	1.93	1.57–2.37	II	Low
Sleep disturbances	Depressive disorders in elderhood	11 (2610)	1.92	1.59–2.33	II	High
Neurotic, stress-related, and somatoform disorders						
None of the factors was supported by class I or II evidence in prospective analysis						
Behavioural syndromes associated with physiological disturbances and physical factors						
None of the factors was supported by class I or II evidence in prospective analysis						
Disorders of adult personality and behaviour						
None of the factors was supported by class I or II evidence in prospective analysis						
Mental retardation						
None of the factors was supported by class I or II evidence in prospective analysis						
Disorders of psychological development						
Maternal SSRI use during pregnancy*	Autism spectrum disorder	3 (19,670)	1.65	1.37–2.00	II	Medium
Maternal overweight pre/during pregnancy	Autism spectrum disorder	4 (>1000)	1.30	1.21–1.40	II	Low
Behavioural and emotional disorders with onset usually occurring in childhood and adolescence						
Maternal pre-pregnancy obesity	ADHD	10 (40,839)	1.63	1.49–1.78	I	Low
Maternal overweight pre/during pregnancy	ADHD	8 (23,484)	1.28	1.20–1.36	I	Low
Maternal paracetamol use during pregnancy*	ADHD	8 (>1000)	1.25	1.17–1.34	I	High
Maternal smoking during pregnancy	ADHD	12 (36,046)	1.60	1.41–1.75	II	High

*AMSTAR* a measurement tool to assess systematic reviews, *ADHD* attention-deficit/hyperactivity disorder, *CI* confidence interval, *CHR-P* clinical high-risk state for psychosis, *RR* risk ratio, *HR* hazard ratio, *SSRIs* selective serotonin-reuptake inhibitors, *T2DM* type 2 diabetes mellitus, *NA* not available.

*Documented or likely confounding by indication; **reversed protective factor. The number of individual studies referred to the number of primary research studies per each meta-analysis included in the umbrella reviews.

#### Quality assessment

Based on the AMSTAR evaluation, nine meta-analyses (45.0%) reported on 12 associations were of high quality, seven meta-analyses (35.0%) reported on nine associations of medium-quality, and four meta-analyses (20.0%) reported on seven associations of low quality (Table 1). The main methodological differences between high/medium and low-quality reviews are described in eResults 1.

### Evidence for the association between risk factors and mental disorders in prospective studies

#### Organic, including symptomatic, mental disorders

The seven class I associations and five risk factors included (Table 1): type 2 diabetes mellitus (T2DM) (vascular dementia, RR = 2.28, 95% CI 1.94–2.66, and Alzheimer’s disease, RR = 1.54, 95% CI 1.39–1.72); depression (any dementia, RR = 1.86, 95% CI 1.61–2.14); depression in elderhood (any dementia, RR = 1.83, 95% CI 1.65–2.03, and Alzheimer’s disease, RR = 1.64, 95% CI 1.40–1.92); low frequency of social contacts (any dementia, RR = 1.57, 95% CI 1.32–1.85); and benzodiazepine use (any dementia, RR = 1.49, 95% CI 1.30–1.72; likely confounding by indication including difficulties with sleep and chronic anxiety with or without depression).

Three class II associations and three risk factors included (Table 1): depression (Alzheimer’s disease, RR = 1.72, 95% CI 1.39–2.13), insufficient physical activity (reversed protective factor, Alzheimer’s disease, RR = 1.62, 95% CI 1.38–1.91; eFigure 1) and T2DM (any dementia, RR = 1.60, 95% CI 1.43–1.79).

#### Mental and behavioural disorders due to psychoactive substance use

No association was supported by class I evidence (Table 1). Only one class II association involved tobacco smoking as a risk factor for opioid use disorder (RR = 2.61, 95% CI = 1.79–3.79).

#### Schizophrenia, schizotypal, and delusional disorders

Only one class I association included (Table 1): clinical high-risk state for psychosis (CHR-P) as a risk factor for any non-organic psychotic disorder (RR = 9.30, 95% CI 4.91–17.66).

Two class II associations included (Table 1): cannabis use (RR = 3.84, 95% CI 2.34–6.29) and childhood adversities (RR = 2.57, 95% CI 1.94–3.40) for schizophrenia spectrum disorders.

#### Mood (affective) disorders

Five class I associations of five risk factors for depressive disorders included (Table 1): sexual dysfunction (RR = 2.49, 95% CI not available), four or five metabolic risk factors (RR = 1.98, 95% CI 1.56–2.53), childhood physical abuse (RR = 1.89, 95% CI 1.70–2.09), job strain (RR = 1.73, 95% CI 1.44–2.06), and obesity (RR = 1.33, 95% CI 1.20–1.47).

Three class II associations included (Table 1): childhood sexual abuse (RR = 2.31, 95% CI 1.72–3.10) and three metabolic risk factors (RR = 1.93, 95% CI 1.57–2.37) as risk factors for depressive disorders, and sleep disturbances as a risk factor for depressive disorders in elderhood (RR = 1.92, 95% CI 1.59–2.33).

#### Neurotic, stress-related and somatoform disorders, behavioural syndromes associated with physiological disturbances and physical factors, disorders of adult personality and behaviour, mental retardation

No class I–II associations/risk factors were identified.

#### Disorders of psychological development

Two class II associations (Table 1) involved two risk factors for autism spectrum disorder: maternal selective serotonin-reuptake inhibitor (SSRI) use during pregnancy (RR = 1.65, 95% CI 1.37–2.00, confounding by indication, such as underlying maternal mental disorders) and maternal overweight pre/during pregnancy (RR = 1.30, 95% CI 1.21–1.40).

#### Behavioural and emotional disorders with onset usually occurring in childhood and adolescence

Three class I associations (Table 1) included three risk factors for ADHD: maternal obesity pre-pregnancy (RR = 1.63, 95% CI 1.49–1.78), maternal overweight pre/during pregnancy (RR = 1.28, 95% CI 1.20–1.36), and maternal paracetamol use during pregnancy (RR = 1.25, 95% CI 1.17–1.34, likely confounding by indication). One class II association (Table 1) involved maternal smoking during pregnancy as a risk factor for ADHD (RR = 1.60, 95% CI 1.41–1.75).

### Global meta-analytic PAF of risk factors for mental disorders

The global meta-analytic PAFs for each mental disorder (in decreasing order of magnitude) with the associated global prevalence (for full prevalence data, see eResults 2 and eTable 2) are presented in Table 2. The PAF of vascular dementia associated with T2DM was 6.73% (95% CI = 5.01–8.72); the PAF of any dementia associated with benzodiazepine use was 5.84% (95% CI = 3.61–8.30), with depression in elderhood 4.30% (95% CI = 3.21–5.60), with T2DM 3.28% (95% CI = 2.35–4.34), and with depression 3.00% (95% CI = 2.13–4.03). The PAF of Alzheimer’s disease associated with insufficient physical activity was 14.60% (95% CI = 9.46–20.52), with depression in elderhood 3.35% (95% CI = 2.06–4.92), with T2DM 2.98% (95% CI = 2.12–3.95), and with depression 2.53% (95% CI = 1.38–3.97). GIF analysis showed that insufficient physical activity should be reduced by 68% to prevent 10% of Alzheimer’s disease (eFigure 5).

**Table 2:** Global meta-analytic PAF for the most robust, potentially modifiable risk factors of mental disorders.

Factor	Mental disorder	Global prevalence	Global PAF	Global PAF 95% lower CI	Global PAF 95% upper CI
Childhood adversities	Schizophrenia spectrum disorders	38.80%	37.84%	26.77%	48.40%
Tobacco smoking	Opioid use disorder	20.47%	24.76%	13.98%	36.49%
Job strain	Depressive disorders	30.00%	17.88%	NA	NA
Insufficient physical activity	Alzheimer’s disease	27.50%	14.60%	9.46%	20.52%
Childhood sexual abuse	Depressive disorders	11.80%	13.40%	7.75%	20.15%
CHR-P	Any non-organic psychotic disorder	1.70%	12.37%	5.37%	25.34%
Maternal paracetamol use during pregnancy*	ADHD	45.00%	10.15%	6.72%	13.74%
Three metabolic risk factors	Depressive disorders	12.00%	10.00%	5.62%	15.95%
Cannabis use	Schizophrenia spectrum disorders	3.80%	9.73%	4.50%	17.30%
Maternal pre-pregnancy obesity	ADHD	16.30%	9.30%	7.36%	11.38%
T2DM	Vascular dementia	5.66%	6.73%	5.01%	8.72%
Childhood physical abuse	Depressive disorders	8.00%	6.60%	5.30%	8.01%
Maternal overweight pre/during pregnancy	Autism spectrum disorder	23.00%	6.47%	4.59%	8.41%
Maternal overweight pre/during pregnancy	ADHD	23.00%	6.02%	4.40%	7.68%
Benzodiazepines use*	Any dementia	12.60%	5.84%	3.61%	8.30%
Four or five metabolic risk factors	Depressive disorders	5.00%	4.69%	2.26%	9.24%
Depression in elderhood	Any dementia	5.41%	4.30%	3.21%	5.60%
T2DM	Any dementia	5.66%	3.28%	2.35%	4.34%
Depression in elderhood	Alzheimer’s disease	5.41%	3.35%	2.06%	4.92%
Depression	Any dementia	3.61%	3.00%	2.13%	4.03%
T2DM	Alzheimer’s disease	5.66%	2.98%	2.12%	3.95%
Obesity	Depressive disorders	8.17%	2.64%	1.63%	3.74%
Depression	Alzheimer’s disease	3.61%	2.53%	1.38%	3.97%
Maternal SSRI use during pregnancy*	Autism spectrum disorder	3.01%	1.93%	1.02%	3.08%
Maternal smoking during pregnancy	ADHD	1.70%	0.98%	0.36%	2.66%

*ADHD* attention-deficit/hyperactivity disorder, *CI* confidence interval, *CHR-P* clinical high-risk state for psychosis, *NA* not available, *PAF* population attributable fraction, *SSRI* selective serotonin-reuptake inhibitors, *T2DM* type 2 diabetes mellitus.

*Documented or likely confounding by indication.

The PAF of opioid use disorder associated with tobacco smoking was 24.76% (95% CI = 13.98–36.49). GIF analysis showed that tobacco smoking should be reduced by 40% to prevent 10% of opioid use disorder cases (eFigure 6).

**Fig. 2:**
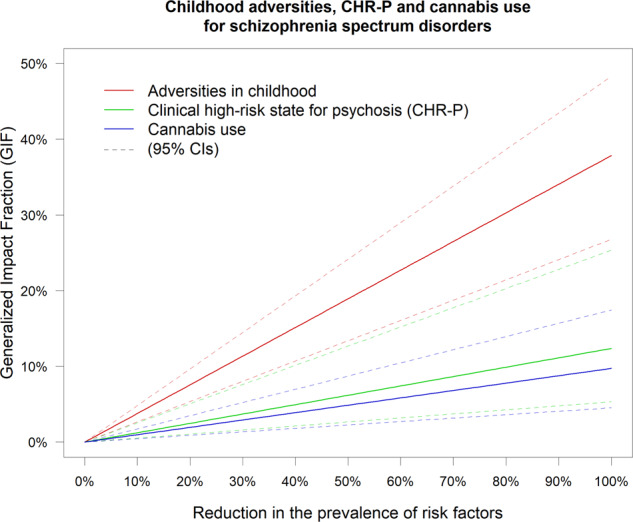
The meta-analytic generalised impact fraction for childhood adversities, clinical high-risk state for psychosis (CHR-P), and cannabis use. Expected proportional reduction in the global incidence of schizophrenia spectrum disorders (Generalized Impact Fraction, GIF) (y-axis) depending on the reduction in the prevalence of risk factors (x-axis).

The PAF of schizophrenia spectrum disorders associated with childhood adversities and cannabis use were 37.84% (95% CI = 26.77–48.40) and 9.73% (95% CI = 4.50–17.30), respectively, while the PAF of any non-organic psychotic disorders associated with the CHR-P was 12.37% (95% CI = 5.37–25.34). GIF analyses showed that childhood adversities should be reduced by 26%, or CHR-P by 81% or cannabis use by 100% to prevent 10% of schizophrenia spectrum disorders (Fig. 2).

The PAF of depressive disorders associations was 17.88% (95% CI = not calculatable) with job strain, 13.40% (95% CI = 7.75–20.15) with childhood sexual abuse, 10.0% (95% CI = 5.62–15.95) with three metabolic risk factors, 6.60% (95% CI = 5.30–8.01) with childhood physical abuse, 4.69% (95% CI = 2.26–9.24) with four or five metabolic risk factors, and 2.64% (95% CI = 1.63–3.74) with obesity. GIF analyses showed that job strain should be reduced by 56%, or childhood sexual abuse by 75% or having three metabolic risk factors by 100% to prevent 10% of depressive disorders (eFigures 7 and 8).

The PAF of autism spectrum disorder associations was 6.47% (95% CI = 4.59–8.41), with maternal overweight pre/during pregnancy and 1.93% (95% CI = 1.02–3.08) with maternal SSRI use during pregnancy.

The PAF of ADHD associations was 10.15% (95% CI = 6.72–13.74) with maternal paracetamol use during pregnancy, 9.30% (95% CI = 7.36–11.38) with maternal pre-pregnancy obesity, 6.02% (95% CI = 4.40–7.68) with maternal overweight pre/during pregnancy, and 0.98% (95% CI = 0.36–2.66) with maternal smoking during pregnancy. The GIF for maternal pre-pregnancy obesity is illustrated in eFigure 9.

**Fig. 3:**
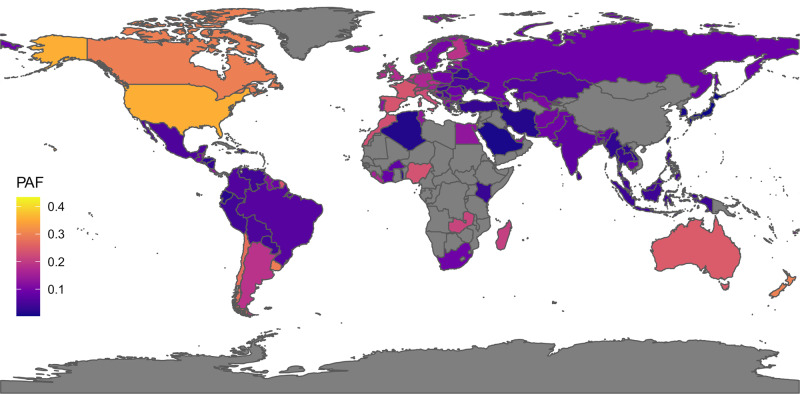
The meta-analytic country-level PAF for cannabis use and schizophrenia spectrum disorders. Expected reduction in the incidence of schizophrenia spectrum disorders (Population Attributable Fraction, PAF) for each country if we could 100% reduce (i.e., eradicate) the prevalence of the risk factor use of cannabis.

Additional sensitivity analyses are reported in the eResults 3, eFigures 5–11, and eTable 6; an illustrative world map of the country-level prevalence of cannabis use is presented in Fig. 3.

## Discussion

We estimated for the first time the global meta-analytic PAFs of 23 robust, potentially modifiable risk factors for mental disorders of class I–II evidence, as published in seven umbrella reviews summarising 295 meta-analyses and 547 associations. These results provide essential epidemiological knowledge that can deconstruct the relative contribution of risk factors to the incidence of mental disorders and inform preventive approaches. By applying the largest literature synthesis and adopting stringent evidence-based classification criteria to rank associations, we identified nine potentially modifiable risk factors with a large PAF (and not confounded by indication) that can be targeted to reduce the global incidence of mental disorders.

The largest global PAF was observed for childhood adversities, which accounted for about two-fifths (38%) of global cases of schizophrenia spectrum disorders. This finding is not surprising, given the relatively high prevalence of adverse childhood experiences, including “toxic stressors”, which can range from bullying experiences to physical or sexual abuse, neglect and even to war crimes [58]. These findings align with psychodynamic theories [59, 60] as well with the more recent social defeat model [61]. Furthermore, a dose-response relationship between childhood adversities and psychotic disorders has been observed [62]. Neurobiologically, childhood adversities are associated with sensitisation of dopamine neurotransmission [63], the key neurotransmitters in psychotic disorders. This study is also the first one to quantify the preventive potential of the clinical high-risk state for psychosis as 12% of global cases of psychosis. As the clinical high risk state for psychosis paradigm was primarily conceived as a targeted and not public health approach, its smaller PAF compared to childhood adversity is expected [3]. Interestingly, the global preventive capacity of other established risk factors, such as cannabis use (10%), was also smaller than childhood adversities and of comparable magnitude as the clinical high-risk state for psychosis. These findings temper recent controversies juxtaposing the utility of preventing psychosis by targeting cannabis abuse or the clinical high-risk state for psychosis, suggesting that both targets hold similar preventive capacity. Notably, as for any other factors in the current study, there was no assumption that these two factors (and therefore PAFs) are independent (e.g., 26% of individuals at clinical high risk for psychosis are also current cannabis users);[64] their combined preventive capacity needs further elucidation by future research [4, 9].

The second-largest PAF was observed for tobacco smoking and opioid use disorder (25%), a finding broadly consistent with established associations between nicotine and opioid dependence [65, 66] and with shared biological underpinnings, extended reinforcement, and cross-tolerance [67]. The third-largest PAF was observed for job strain and depression (18%), which emerges as a core modifiable target among working adults [68]. This result aligns with the substantial and widespread impact of job strain on other physical health outcomes, including metabolic syndrome [69], coronary heart disease [70], diabetes [71], stroke [72], musculoskeletal pain [73] and even mortality [74].

Interestingly, one specific type of childhood adversity (sexual abuse) emerged also as a preventive target for depressive disorders (13%), highlighting its potential transdiagnostic capacity, which could allow preventing multiple mental disorders, and better justify the cost of any preventive intervention.

Overall, the public health implication of these findings is to recommend prioritising resources to reduce a small risk among many (and ideally across different mental disorders) rather than vice versa [75]. This approach could be further enhanced by simultaneously targeting mental and physical health domains, maximising the resulting preventive potential. For example, we identified three preventive targets pertaining to physical health domains (PAFs from 10-25%), which have been associated with neuroinflammatory mechanisms [76]: three metabolic factors and depression, insufficient physical activity and Alzheimer’s disease, tobacco smoking and opioid use disorders.

Notably, while we primarily focused on global PAFs, the prevalence of these factors varies profoundly across different countries and demographic groups. To explore this issue, we performed sensitivity analyses using specific prevalence data. The largest PAF was confirmed for childhood adversities, with comparable magnitude across high-, middle- and low-income countries. Country-level PAFs were highly variable for tobacco smoking, job strain, and cannabis use. The PAF for tobacco smoking and opioid use disorders was higher in Europe versus the USA and in men versus women, while the PAF for insufficient physical activity and Alzheimer’s disease tended to be reduced in low-income countries. Some PAFs were particularly marked in specific groups: childhood sexual abuse and depressive disorders among women, four or five metabolic risk factors and depressive disorders in adults >70 years, T2DM and vascular dementia or Alzheimer’s disease in adults >70 years. This great variation of PAFs may indicate a complex interplay of sociodemographic, health, and economic factors, which future research should better address.

Although our results for factors with the largest PAFs are derived from high- to medium-quality meta-analyses (with few exceptions), this study has some important limitations. First, while the term “attributable” in the PAF usually has a causal interpretation [77], there are no clear aetiopathological factors identified for mental disorders but only statistical associations. As most associations of risk factors with mental disorders typically emerged from observational cohort studies, which are liable to confounding [30], the estimated PAF effect is not adjusted for all possible confounders (and the risk factors are not necessarily independent and probably intercorrelated, see eLimitation). However, we did carefully identify potential confounding by indication. Because of these limitations the PAFs reported in this study should be distinguished from the aetiologic fraction [78]. Another limitation is that there are no established cut-offs to distinguish between large and small PAFs. Furthermore, the PAF is a static measure that assumes that removing an exposure does not affect the person–time at risk and onwards effects, which may not be true for some factors [30], particularly for those exerting their effect during early neurodevelopmental stages. A transmission PAF (tPAF) [79, 80] has been suggested to mathematically estimate onward transmission of the potential long-term preventive gains [75]. Possible caveats that may result in over- or underestimation of the prevalence estimates for several risk factors should also be considered. Reliable population-level data were not always available, and we were unable to calculate the global PAF for some factors (low frequency of social contacts, sexual dysfunction, sleep disturbances) and the specific PAFs for several factors. Future research should address the global and specific prevalence of these factors. Finally, while this study focuses on the hypothetical preventive capacity of robust non-primarily genetic targets; the real-world effectiveness of specific preventive interventions targeting these factors should be demonstrated and appraised by separate analyses.

## Conclusions

Addressing several potentially modifiable risk factors, in particular childhood adversities, can potentially reduce the global population-level incidence of mental disorders. Future research should prioritise these preventive targets.

## Supplementary information

Supplementary information

## Data Availability

The meta-analytic data are published and freely accessible.
