# Supplementary material for: Global population attributable fraction of potentially modifiable risk factors for mental disorders: a meta-umbrella systematic review
Source: Mol Psychiatry. 2022 Apr 28;27(8):3510–9. doi: 10.1038/s41380-022-01586-8 (PMC9708560; doi:10.1038/s41380-022-01586-8)
Supplement: Supplementary file 1 — Supplementary information [file 41380_2022_1586_MOESM1_ESM.docx]

**Supplementary information**

Dragioti, Radua et al., 2021. Global Population Attributable Fraction of Potentially Modifiable Risk Factors for Mental Disorders: A Meta-Umbrella Systematic Review

[eMethods 1. PRISMA 2020 abstract checklist and PRISMA checklist 3](#_Toc97817449)

[eBox1. Full search strategy per database 7](#_Toc97817450)

[eMethods 2. ICD-10 diagnostic blocks 7](#_Toc97817451)

[eMethods 3. Additional computations for global prevalence data 7](#_Toc97817452)

[eMethods 4. Sensitivity analyses 8](#_Toc97817453)

[eResults 1. Main methodological differences between high/medium and low-quality reviews 9](#_Toc97817454)

[eResults 2. Prevalence data employed in the meta-analytic PAF analysis 9](#_Toc97817455)

[eResults 3. Additional sensitivity meta-analytic PAF analyses 10](#_Toc97817456)

[eLimitation. The interplay between investigated risk factors 11](#_Toc97817457)

[eTable 1. Operationalisation of risk factors as provided by each meta-analysis included in umbrella reviews 12](#_Toc97817458)

[eTable 2. Prevalence estimates data employed in the study 14](#_Toc97817459)

[eTable 3. Characteristics of the studies included for prevalence estimates if GBD 2019 prevalence data were not available 17](#_Toc97817460)

[eTable 4. Articles excluded after full-text revision, with reasons 20](#_Toc97817461)

[eTable 5. Overall characteristics of the umbrella reviews included in the current study 23](#_Toc97817462)

[eTable 6. Specific PAFs for the most robust risk factors of mental disorders 24](#_Toc97817463)

[eFigure 1. Forest plot for the reversed factor of high physical activity to insufficient physical activity (Beckett et al. 2015)^20^ 26](#_Toc97817464)

[eFigure 2. Meta-analysis of the proportion of four or five metabolic risk factors 27](#_Toc97817465)

[eFigure 3. Meta-analysis of the proportion of three metabolic risk factors 28](#_Toc97817466)

[eFigure 4. Meta-analysis of the proportion of maternal paracetamol use during pregnancy (Zafeiri et al. 2021)^1^ 29](#_Toc97817467)

[eFigure 5. The meta-analytic Generalized Impact Fraction for insufficient physical activity 30](#_Toc97817468)

[eFigure 6. The meta-analytic Generalized Impact Fraction for tobacco smoking 31](#_Toc97817469)

[eFigure 7. The meta-analytic Generalized Impact Fraction for job strain 32](#_Toc97817470)

[eFigure 8. The meta-analytic Generalized Impact Fraction for sexual abuse in childhood (a) and three metabolic risk factors (b) 33](#_Toc97817471)

[eFigure 9.The meta-analytic Generalized Impact Fraction for maternal pre-pregnancy obesity 34](#_Toc97817472)

[eFigure 10. The meta-analytic country-level PAF for tobacco smoking and opioid use disorder 35](#_Toc97817473)

[eFigure 11. The meta-analytic country-level PAF for job strain and depressive disorders 36](#_Toc97817474)

[eReferences 37](#_Toc97817475)

[References of excluded umbrella reviews with reasons 40](#_Toc97817476)

# eMethods 1. PRISMA 2020 abstract checklist and PRISMA checklist

| **Section and Topic** | **Item #** | **Checklist item** | **Reported (Yes/No)** |
| --- | --- | --- | --- |
| **TITLE** | | |  |
| Title | 1 | Identify the report as a systematic review. | Yes |
| **BACKGROUND** | | |  |
| Objectives | 2 | Provide an explicit statement of the main objective(s) or question(s) the review addresses. | Yes |
| **METHODS** | | |  |
| Eligibility criteria | 3 | Specify the inclusion and exclusion criteria for the review. | Yes |
| Information sources | 4 | Specify the information sources (e.g., databases, registers) used to identify studies and the date when each was last searched. |  |
| Risk of bias | 5 | Specify the methods used to assess risk of bias in the included studies. | Yes |
| Synthesis of results | 6 | Specify the methods used to present and synthesise results. | Yes |
| **RESULTS** | | |  |
| Included studies | 7 | Give the total number of included studies and participants and summarise relevant characteristics of studies. | Yes |
| Synthesis of results | 8 | Present results for main outcomes, preferably indicating the number of included studies and participants for each. If meta-analysis was done, report the summary estimate and confidence/credible interval. If comparing groups, indicate the direction of the effect (i.e. which group is favoured). | Yes |
| **DISCUSSION** | | |  |
| Limitations of evidence | 9 | Provide a brief summary of the limitations of the evidence included in the review (e.g. study risk of bias, inconsistency and imprecision). | Yes |
| Interpretation | 10 | Provide a general interpretation of the results and important implications. | Yes |
| **OTHER** | | |  |
| Funding | 11 | Specify the primary source of funding for the review. | Yes |
| Registration | 12 | Provide the register name and registration number. | Yes, registration number not available |

PRISMA 2020 checklist

| **Section and Topic** | **Item #** | **Checklist item** | **Location where item is reported** |
| --- | --- | --- | --- |
| **TITLE** | | |  |
| Title | 1 | Identify the report as a systematic review. | 1 |
| **ABSTRACT** | | |  |
| Abstract | 2 | See the PRISMA 2020 for Abstracts checklist. | Page 2 in the supplement |
| **INTRODUCTION** | | |  |
| Rationale | 3 | Describe the rationale for the review in the context of existing knowledge. | 5 |
| Objectives | 4 | Provide an explicit statement of the objective(s) or question(s) the review addresses. | 5-6 |
| **METHODS** | | |  |
| Eligibility criteria | 5 | Specify the inclusion and exclusion criteria for the review and how studies were grouped for the syntheses. | 6 |
| Information sources | 6 | Specify all databases, registers, websites, organisations, reference lists and other sources searched or consulted to identify studies. Specify the date when each source was last searched or consulted. | 6 |
| Search strategy | 7 | Present the full search strategies for all databases, registers and websites, including any filters and limits used. | eBox1 |
| Selection process | 8 | Specify the methods used to decide whether a study met the inclusion criteria of the review, including how many reviewers screened each record and each report retrieved, whether they worked independently, and if applicable, details of automation tools used in the process. | 7 |
| Data collection process | 9 | Specify the methods used to collect data from reports, including how many reviewers collected data from each report, whether they worked independently, any processes for obtaining or confirming data from study investigators, and if applicable, details of automation tools used in the process. | 7 |
| Data items | 10a | List and define all outcomes for which data were sought. Specify whether all results that were compatible with each outcome domain in each study were sought (e.g. for all measures, time points, analyses), and if not, the methods used to decide which results to collect. | 7 |
|  | 10b | List and define all other variables for which data were sought (e.g. participant and intervention characteristics, funding sources). Describe any assumptions made about any missing or unclear information. | NA |
| Study risk of bias assessment | 11 | Specify the methods used to assess risk of bias in the included studies, including details of the tool(s) used, how many reviewers assessed each study and whether they worked independently, and if applicable, details of automation tools used in the process. | 8 |
| Effect measures | 12 | Specify for each outcome the effect measure(s) (e.g. risk ratio, mean difference) used in the synthesis or presentation of results. | 7-8 |
| Synthesis methods | 13a | Describe the processes used to decide which studies were eligible for each synthesis (e.g. tabulating the study intervention characteristics and comparing against the planned groups for each synthesis (item #5)). | 7-8 |
|  | 13b | Describe any methods required to prepare the data for presentation or synthesis, such as handling of missing summary statistics, or data conversions. | 7-8 |
|  | 13c | Describe any methods used to tabulate or visually display results of individual studies and syntheses. | 7-9 |
|  | 13d | Describe any methods used to synthesize results and provide a rationale for the choice(s). If meta-analysis was performed, describe the model(s), method(s) to identify the presence and extent of statistical heterogeneity, and software package(s) used. | NA |
|  | 13e | Describe any methods used to explore possible causes of heterogeneity among study results (e.g. subgroup analysis, meta-regression). | NA |
|  | 13f | Describe any sensitivity analyses conducted to assess robustness of the synthesized results. | Suppl. methods |
| Reporting bias assessment | 14 | Describe any methods used to assess risk of bias due to missing results in a synthesis (arising from reporting biases). | NA |
| Certainty assessment | 15 | Describe any methods used to assess certainty (or confidence) in the body of evidence for an outcome. | NA |
| **RESULTS** | | |  |
| Study selection | 16a | Describe the results of the search and selection process, from the number of records identified in the search to the number of studies included in the review, ideally using a flow diagram. | Figure 1 |
|  | 16b | Cite studies that might appear to meet the inclusion criteria, but which were excluded, and explain why they were excluded. | 9, Figure 1, eTable 1 |
| Study characteristics | 17 | Cite each included study and present its characteristics. | 9, Table 1 |
| Risk of bias in studies | 18 | Present assessments of risk of bias for each included study. | Table 2, eTable3 |
| Results of individual studies | 19 | For all outcomes, present, for each study: (a) summary statistics for each group (where appropriate) and (b) an effect estimate and its precision (e.g. confidence/credible interval), ideally using structured tables or plots. | Table 2 |
| Results of syntheses | 20a | For each synthesis, briefly summarise the characteristics and risk of bias among contributing studies. | NA |
|  | 20b | Present results of all statistical syntheses conducted. If meta-analysis was done, present for each the summary estimate and its precision (e.g. confidence/credible interval) and measures of statistical heterogeneity. If comparing groups, describe the direction of the effect. | NA |
|  | 20c | Present results of all investigations of possible causes of heterogeneity among study results. | NA |
|  | 20d | Present results of all sensitivity analyses conducted to assess the robustness of the synthesized results. | eResults |
| Reporting biases | 21 | Present assessments of risk of bias due to missing results (arising from reporting biases) for each synthesis assessed. | NA |
| Certainty of evidence | 22 | Present assessments of certainty (or confidence) in the body of evidence for each outcome assessed. | NA |
| **DISCUSSION** | | |  |
| Discussion | 23a | Provide a general interpretation of the results in the context of other evidence. | 11-12 |
|  | 23b | Discuss any limitations of the evidence included in the review. | 13 |
|  | 23c | Discuss any limitations of the review processes used. | 13 |
|  | 23d | Discuss implications of the results for practice, policy, and future research. | 13 |
| **OTHER INFORMATION** | | |  |
| Registration and protocol | 24a | Provide registration information for the review, including register name and registration number, or state that the review was not registered. | 6 |
|  | 24b | Indicate where the review protocol can be accessed, or state that a protocol was not prepared. | 6 |
|  | 24c | Describe and explain any amendments to information provided at registration or in the protocol. | NA |
| Support | 25 | Describe sources of financial or non-financial support for the review, and the role of the funders or sponsors in the review. | 8 |
| Competing interests | 26 | Declare any competing interests of review authors. | 14 |
| Availability of data, code and other materials | 27 | Report which of the following are publicly available and where they can be found: template data collection forms; data extracted from included studies; data used for all analyses; analytic code; any other materials used in the review. | Suppl. material |

# eBox1. Full search strategy per database

| **Web of Science** |
| --- |
| ALL=((umbrella review) AND ("risk" OR "protect*")) |
| **PubMed** |
| ("umbrella"[All Fields] OR "umbrellas"[All Fields]) AND ("review"[Publication Type] OR "review literature as topic"[MeSH Terms] OR "review"[All Fields]) AND ("risk"[All Fields] OR "protect*"[All Fields]) |
| **Ovid/PsycINFO** |
| (umbrella review) AND ("risk" OR "protect*") |
| **Cochrane** |
| (umbrella review):ti,ab,kw AND (risk):ti,ab,kw OR (protective):ti,ab,kw |

# eMethods 2. ICD-10 diagnostic blocks

Mental disorders were stratified by using the corresponding ICD-10 diagnostic blocks: organic, including symptomatic, mental disorders; mental and behavioural disorders due to psychoactive substance use; schizophrenia, schizotypal and delusional disorders; mood (affective) disorders; neurotic, stress-related and somatoform disorders; behavioural syndromes associated with psychological disturbances and physical factors; disorders of adult personality and behaviour; mental retardation; disorders of psychological development; and behavioural and emotional disorders with onset usually occurring in childhood and adolescence. We did not use ICD-11 because it is still not captured by published/existing literature.

# eMethods 3. Additional computations for global prevalence data

Whenever a prevalence study or meta-analysis provided prevalence data per country (e.g., for maternal paracetamol use during pregnancy from 13 countries, i.e., Australia, Denmark, Ethiopia, France, Germany, Italy, Netherlands, Pakistan, Saudi Arabia, Serbia, UK, USA, and United Arab Emirates)^1^ but not an overall estimate, we calculated the overall prevalence estimate using the Metaprop module in Stata.^2^ The 95% CIs of the pooled prevalence rate were calculated using the cimethod (exact) and the Freeman-tukey double arcsine transformation (ftt command), which computes the weighted pooled estimate and performs the back-transformation on the pooled estimate.^2^ When a robust global prevalence could not be identified via searching, we also extracted data from the original meta-analyses included in umbrella reviews for the specific risk factors to compute the prevalence. If no valid prevalence meta-analyses matching the operationalisation of the risk factors were identified, we did not estimate the PAF and acknowledged the missingness of prevalence data. When CIs were not reported, we calculated them using the cii proportions immediate command in Stata (StataCorp. 2017. Stata Statistical Software: Release 17. College Station, TX). Overall, we performed meta-analyses of proportions for the following factors: maternal paracetamol use during pregnancy)^1^, four or five metabolic risk factors^3^, and three metabolic risk factors^3^. The forest plots for those prevalence meta-analyses are presented in eFigures 2-4. Only for one study of Kessler et al. 2010^4^, we used the proportions immediate command to calculate the CIs for the reported prevalence. For the global job strain prevalence, CIs could not be estimated due to the lack of the total number of individuals.

# eMethods 4. Sensitivity analyses

We found high variability in the prevalence of risk factors identified by our review across different regions and demographic groups. To control for this issue, we conducted sensitivity analyses using specific prevalence data. Whenever feasible, we additionally extracted specific prevalence data, which included:

1. country-level prevalence (for factors with a global high PAF and not confounded by indication)*
2. regional prevalence for Europe
3. regional prevalence for the USA
4. prevalence in high-income countries
5. prevalence in middle-income countries
6. prevalence in low-income countries
7. prevalence in males
8. prevalence in females
9. prevalence in young people (variably defined)
10. prevalence in older adults (≥70 years)

* this analysis was not planned in the original protocol.

We then used these prevalence data to estimate the specific PAFs (analyses b to j). For analysis (a), we produced a global map of country-level PAFs.

# eResults 1. Main methodological differences between high/medium and low-quality reviews

The main methodological differences between high/medium and low-quality reviews were the inadequate performance in low quality reviews across the three of the seven critical AMSTAR 2 domains, i.e., reviews established an a priori protocol and justified deviations from the protocol (item 2), implemented a comprehensive literature search strategy (item 4) and listed excluded studies and justified the exclusions (item 7). In addition, in low quality reviews the performance was also unsatisfactory among three of the nine non-critical domains i.e., explained the selection of study designs for inclusion (item 3), described included studies in adequate details (item 8) and reported sources of funding among individual studies included (item 10).”

# eResults 2. Prevalence data employed in the meta-analytic PAF analysis

We preferably used prevalence estimates from GBD 2019, followed by GBD 2015 data if the data needed could not be retrieved in the 2019 GBD (four risk factors, eTable 3). For the remaining factors both the 2019 and 2015 GBD provided only adjusted age prevalence estimates or different operationalisations of factors that did not match those included in the umbrella reviews. Therefore we further used prevalence estimates from one WHO global report (one risk factor), one report from the United Nations Office on Drugs and Crime (UNDOC; one risk factor), one report from the Organisation for Economic Co-operation and Development (OECD; one risk factor), one report from the Centers for Disease Control and Prevention (CDC; one risk factor), seven meta-analyses (nine risk factors), one systematic review (one risk factor), and four population-based studies (six risk factors). These data are detailed in eTables 3-4 (see, eReferences 1,3,4 and 31-47). GBD 2019 and 2015 global prevalence ranged from 8.17% for obesity to 3.61% for depression; the WHO global prevalence for tobacco smoking was 20.47%; the UNDOC prevalence for cannabis use was 3.80%; the OECD prevalence for job strain was 30.00%; meta-analytic global prevalence (>37,822,312 participants) ranged from 38.80% for adversities in childhood to 1.70% for clinical high-risk state for psychosis and maternal smoking during pregnancy; the systematic review (48,613 participants) provided global prevalence data of 46.00% for maternal paracetamol use during pregnancy; the population-based studies (up to 2,044,155 participants) global prevalence ranged from 38.80% for childhood adversities to 8.00% for childhood physical abuse. The median quality assessment score of the population-based studies was 7 (interquartile range: 7-8), while the quality of included prevalence meta-analyses/systematic reviews according to AMSTAR was high in three, medium in three, and low in two meta-analyses/systematic reviews. Prevalence data were not available for three factors (low frequency of social contacts, sexual dysfunction, and sleep disturbances). Notably, we did not use PAFs already calculated in GBD 2019, because we were interested in meta -analytic PAF estimates.

# eResults 3. Additional sensitivity meta-analytic PAF analyses

The specific meta-analytic PAFs (in decreasing order of magnitude) are detailed in eTable 5 and in the eFigures 5-11. Country-level prevalence data were available for tobacco smoking, job strain (eFigures 10,11), and cannabis use (Figure 3 in the main text). For tobacco smoking, the highest PAF was found in Kiribati (43.08%) and the lowest in Honduras (3.12%). For job strain, the highest PAF was found in Greece (25.90%) and the lowest in Norway (9.16%). For cannabis use, the highest PAF was found in Israel (43.39%) and the lowest in Japan (0.28%). High PAFs were found for childhood adversities associated with schizophrenia spectrum disorders in high- (37.59%), middle- (37.90%), and low-income countries (38.02%). High PAFs were found for tobacco smoking associated with opioid use disorder in Europe (32.46%), USA (18.37%), high- (25.77%), middle- (23.86%), and low-income countries (15.26%), men (34.45%), and women (26.99%). High PAFs were found for insufficient physical activity associated with Alzheimer's disease in Europe (12.70%), USA (13.64%), high- (18.62%) and middle-income countries (13.92%), men (12.70%), and women (16.46%). High PAF was found for childhood sexual abuse associated with depressive disorders in women (19.78%). High PAFs were found for clinical high-risk state for psychosis associated with any non-organic psychotic disorder in Europe (13.00%), USA (21.51%), and high-income countries (13.00%). High PAFs were found for cannabis use associated with schizophrenia spectrum disorders in Europe (13.26%) and USA (19.98%). High PAFs were found for type 2 diabetes mellitus associated with vascular dementia in Europe (9.01%), USA (9.28%), high- income countries (10.25%), and in adults >70 years (22.84%). High PAF was found for four or five metabolic risk factors associated with depressive disorders in adults >70 years (35.14%). Finally, high PAFs were found for type 2 diabetes mellitus and for depression associated with any dementia in adults >70 years (12.22% and 11.19% respectively).

# eLimitation. The interplay between investigated risk factors

Notably, the risk factors investigated are unlikely to be entirely uncorrelated; future research should consider the full correlation matrix between risk factors and identify their cumulative impact. However, these analyses are possible only in the context of large-scale studies that measure the concurrent exposure of numerous risk factors. The list of those identified in the present investigation may provide an initial set of exposures to be measured in multivariable approaches by future studies.

# eTable 1**.** Operationalisation of risk factors as provided by each meta-analysis included in umbrella reviews

| Cannabis use^12^ | Five different levels of cannabis exposure, i.e., never used (comparison group), used fewer than 5 times in a lifetime, 5-20 times, 21-60 times, and >60 times (ever user groups) at any age |
| --- | --- |
| Depression^13^ | Depressive disorders in patients at any age by valid diagnostic measurements compared to individuals with no mental disorder |
| Depression in elderhood^14^ | “Late-life” depression by valid diagnostic measurements in older adults compared to not diseased |
| Obesity^3^ | BMI ≥ 30kg/m^2^ at any age |
| Tobacco smoking^15^ | Current smokers versus non-smokers at any age |
| Type 2 diabetes mellitus^16^ | Adult patients (>40 years of age) with diabetes mellitus type 2 compared with non‐diabetes patients |
| Job strain^17^ | As the combination of high demands and low control from the validated job strain scale (IPD-Work Consortium) compared to those without job strain in adults (>18 years of age) |
| Sexual dysfunction^18^ | A syndrome that covers lack or loss of sexual desire, sexual aversion and lack of sexual enjoyment, failure of genital response (erectile dysfunction in men and vaginal dryness or failure of lubrication in women), orgasmic dysfunction, premature ejaculation, vaginismus in women, and dyspareunia (or pain during sexual intercourse) in people aged from 20 to 57 years |
| Sleep disturbances^19^ | Having insomnia, poor sleep quality, and complaints of insomnia in adults (≥50 years of age) |
| Insufficient physical activity ^20^ | Physically active older adults compared to their non-active or insufficient active counterparts |
| Four or five metabolic risk factors^3^ | Having four or five of the following markers: high blood pressure (>130mmHg systolic or >85mmHg diastolic), high triglycerides (>1.7mmol/L), low HDL cholesterol (<1.03mmol/L in men, <1.29mmol/L in women), impaired glucose metabolism (glycated haemoglobin HA1c > 6%), and high C-reactive protein (CRP>3.0mg/dL) at any age |
| Three metabolic risk factors^3^ | Having at least three of the following markers: high blood pressure (>130mmHg systolic or >85mmHg diastolic), high triglycerides (>1.7mmol/L), low HDL cholesterol (<1.03mmol/L in men, <1.29mmol/L in women), impaired glucose metabolism (glycated haemoglobin HA1c > 6%), and high C-reactive protein (CRP>3.0mg/dL) at any age |
| Low frequency of social contacts^21^ | Lower frequency of social contact (e.g., visiting or receiving phone calls from friends, children or other relatives) compared to older adults with a higher frequency of social contacts |
| Maternal overweight pre/during pregnancy^22,23^ | BMI = 25–29.9 kg/m^2^ before or during pregnancy by medical records or self-reported |
| Maternal pre-pregnancy obesity^23^ | BMI ≥ 30kg/m^2^ before pregnancy |
| Maternal SSRI use during pregnancy^24^ | Maternal exposure to SSRI during pregnancy by medical recodes and registries. |
| Maternal smoking during pregnancy^25^ | Light and severe smoking during pregnancy by interviews or according to related registers |
| Clinical high-risk state for psychosis^26^ | An established psychometric instrument for the clinical high-risk for psychosis state |
| Childhood sexual abuse ^27^ | Exposing or involvement of a child in sexual activities (fondling children’s genitals, oral-genital contact, using  objects for vaginal or anal penetration, vaginal or anal intercourse) and or non-physical contact (showing children pornography or using children to produce pornographic material, and exposing children to sexual intercourse) before 18 years |
| Maternal paracetamol use during pregnancy^28^ | Maternal exposure to acetaminophen or paracetamol during pregnancy |
| Childhood adversities ^29^ | Any type of childhood adversity, including sexual abuse, physical abuse, emotional/psychological abuse, neglect, bullying, and parental death that occurred prior to the age of 18 |
| Childhood physical abuse ^27^ | Physical harm that a child undergoes from their caregiver, including hitting, punching, kicking, biting, burning, poisoning, and suffocating, before 18 years |
| Benzodiazepines use^30^ | Ever use of benzodiazepines versus never in adults >18 years by prescription records or interview |

# eTable 2. Prevalence estimates data employed in the study

| **Factor** | **Source of prevalence data (type, age)** | **Global prevalence % (95%CI)** | **Prevalence in Europe %** | **Prevalence in USA %** | **Prevalence in high-income countries %** | **Prevalence in middle-income countries %** | **Prevalence low-income countries %** | **Prevalence in men %** | **Prevalence in women %** | **Prevalence in young people 15-49 years %** | **Prevalence in adults ≥70 years %** |
| --- | --- | --- | --- | --- | --- | --- | --- | --- | --- | --- | --- |
| Cannabis use^31,32^ | UNDOC (point prevalence, 1-year, age range 15 to 64 years) ^31^ | 3.80 (2.70-4.90) | 5.39 | 8.80 | - | - | - | - | - | - | - |
| Depression^33^ | GBD 2019 (point prevalence 1-year, all ages) | 3.61 (3.25-4.01) | 4.25 | 3.89 | 4.05 | 3.46 | 3.23 | 2.81 | 4.42 | 4.26 | 5.41 |
| Depression in elderhood^33^ | GBD 2019(point prevalence 1-year, above 70 years old) | 5.41 (4.52-6.40) | 5.42 | 4.32 | 4.08 | 5.52 | 7.66 | 4.44 | 6.17 | - | 5.41 |
| Obesity^34^ | GBD 2015 (point prevalence 1-year, all ages) | 8.17 (8.02-8.36) | 16.82 | 24.82 | 7.87 | 2.89 | 1.27 | 10.0 | 14.6 | - | - |
| Tobacco smoking | WHO 2019 (point prevalence 1-year, above 15 years old)^35^; Jafari et al. 2021 (lifetime, adolescent girls/students of the school)^36^ | 20.47 (20.46-20.47) | 29.90 | 14.0^37^ | 21.6 | 19.5 | 11.2 | 32.7 | 23.0^36^ | - | - |
| Type 2 diabetes mellitus^33^ | GBD 2019 (point prevalence 1 year, all ages) | 5.66 (5.20-6.17) | 7.66 | 8.02 | 8.95 | 5.81 | 2.54 | 5.83 | 5.48 | 3.06 | 23.20 |
| Job strain^38^ | OECD 2015 ( point prevalence 1 year, 15 to 64 years) ^38^ | 30.0* | - | - | - | - | - | - | - | - | - |
| Insufficient physical activity | Guthold et al. 2018 (point prevalence 1 year, above 18 years old)^39^  CDC 2018 (point prevalence1 year, adults) ^40^ | 27.50 (25.0-32.0)^39^ | 23.4^39^ | 25.4^40^ | 36.8^39^ | 26.0^39^ | 16.2^39^ | 23.4^39^ | 31.7^39^ | - | - |
| Four or five metabolic risk factors | Jokela et al. 2014 (2-4 years,15 to 105 years old)^3^  Kuk & Arden 2010 (1 year, above 18 years old)^41^ | 5.0 (3.0-9.0)** New meta-analysis (eFigure 2)^3^ | - | -- | - | - | - | 8.0^41^ | 9.2^41^ | - | 55.0^41^ |
| Three metabolic risk factors | Jokela et al. 2014 (2-4 years,15 to 105 years old)^3^  Kuk & Arden 2010 (1 year, above 18 years old) ^41^ | 12.0 (8.0-17.0)** New meta-analysis (eFigure 3)^3^ | - | - | - | - | - | 4.8^41^ | 4.2^41^ | - | - |
| Maternal overweight pre/during pregnancy^42^ | Martinez-Hortelano et al. 2020 (8 years, maternal age from 23.6 to 33.0 years)^42^ | 23.0 (22.30-23.70) | 19.5 | 26.8 | - | - | - | NA | 23.0 | - | NA |
| Maternal pre-pregnancy obesity^42^ | Martinez-Hortelano et al. 2020(8 years, maternal age from 23.6 to 33.0 years)^42^ | 16.30 (15.40-17.30) | 9.1 | 17.6 | - | - | - | NA | 16.30 | - | NA |
| Maternal SSRI use during pregnancy^43^ | Molenaar et al. 2020 (11 years, maternal age from 10 to 55 years)^43^ | 3.01 (2.29-3.74) | 1.64 | 5.46 | - | - | - | NA | 3.0 | - | NA |
| Maternal smoking during pregnancy | Lange et al. 2018 (30 years, women during pregnancy)^44^  Jafari et al. 2021 (lifetime, women during pregnancy)^36^ | 1.70 (0.00-4.50)^44^ | 8.1^44^ | 5.9^44^ | - | - | - | NA | 32.0^36^ | - | NA |
| Clinical high-risk state for psychosis^45^ | Salazar de Pablo et al. 2021 (1-5 year,14 to 24 years) ^45^ | 1.7 (1.0-2.9) | 1.8 | 3.3 | 1.8 | 1.2 | - | - | - | - | - |
| Childhood sexual abuse ^a^ | Stoltenborgh et al. 2011 (1-18 year, prior to the age of 18 )^46^  Kessler et al. 2010 (lifetime, prior to the age of 18)^4^ | 11.80 (10.0-13.80)^46^ | - | - | 2.4^4^ | 0.6^4^ | 1.5^4^ | 7.6^46^ | 18.0^46^ | - | - |
| Maternal paracetamol use during pregnancy^1^ | Zafeiri et al. 2021(12 years, women during pregnancy) ^1^ | 45.0 (36.0-53.0) New meta-analysis (eFigure 4)** | - | - | - | - | - | NA | - | - | NA |
| Childhood adversities^4^ | Kessler et al. 2010 (lifetime, prior to the age of 18)^4^ | 38.80 (37.14-40.48)*** | - | - | 38.4 | 38.9 | 39.1 | - | - | - | - |
| Childhood physical abuse^4^ | Kessler et al. 2010 (lifetime, prior to the age of 18)^4^ | 8.0 (7.96-8.03) | - | - | 5.3 | 10.8 | 9.0 | - | - |  | - |
| Benzodiazepines use^47^ | Maust et al. 2019 (point prevalence 1-year, above 18 years old)^47^ | 12.60 (12.20-12.90) | - | 12.60 | - | - | - | - | - | - | - |

^a^ for sexual abuse we did not use GBD 2019 data because the age of occurrence was under 15, CI=confidence interval, SSRI = Selective serotonin reuptake inhibitors, NA= Not applicable *CIs not predictable, **the overall prevalence for those factors was computed by us ( see eFigures ***CIs estimated by cci proportions immediate stata command from the prevalence provided in the study of Kessler et al. 2010^4^

# eTable 3. Characteristics of the studies included for prevalence estimates if GBD 2019 prevalence data were not available

| **Factor** | | **Study (ref)** | **Study design** | **No of included studies/years review** | **N total** | **Study quality** |
| --- | --- | --- | --- | --- | --- | --- |
| Cannabis use | (Global, Europe and USA) | UNDOC, 2017^31^ | United Nations Office on Drugs and Crime, World Drug Report 2017 | NA/2017 | NR | NA |
| Tobacco smoking | (Global, Europe, high, middle, low-income countries, and men) | WHO 2019^35^ | WHO global report | NA/2000-2015 | NR | NA |
|  | (USA) | Cornelius et al., 2020^37^ | NHIS U.S. annual report | NA/2019 | 31 997 | NA |
|  | (women) | Jafari et al. 2021^36^ | Meta-analysis | 109/January 2010 to April 2020 | 18 290 793 | Medium |
| Job strain | (Global) | OECD 2015 ^38^ | Organisation for Economic Cooperation and Development (OECD) report* | 7 international surveys/2015 | NR | NA |
| Four or five metabolic risk factors | (Global) | Jokela et al. 2014 ^3^ | Individual participant meta-analysis | 8/ Inter-University Consortium for Political and Social Research and the Economic and Social Data Service | 30 337 | Low |
|  | (Men and women, older adults) | Kuk & Arden 2010 ^41^ | Population-based | NHANES III with follow-up through 31 December 2006 | 6 024 | 7/10 |
| Three metabolic risk factors | (Global) | Jokela et al. 2014 ^3^ | Individual participant meta-analysis | 8/ Inter-University Consortium for Political and Social Research and the Economic and Social Data Service | 30 337 | Low |
|  | (Men and women) | Kuk & Arden 2010 ^41^ | Population-based | NHANES III with follow-up through 31 December 2006 | 6 024 | 7/10 |
| Maternal overweight pre/during pregnancy | (Global, Europe, USA, and women) | Martinez-Hortelano et al 2020^42^ | Meta-analysis | 63/2009 to May 2018 (after the 2009 IOM guidelines) | 1 416 915 | Medium |
| Maternal pre-pregnancy obesity | (Global, Europe, USA, and women) | Martinez-Hortelano et al 2020^42^ | Meta-analysis | 63/2009 to May 2018 (after the 2009 IOM guidelines) | 1 416 915 | Medium |
| Maternal SSRI use during pregnancy | (Global, Europe, USA, and women) | Molenaar et al. 2020^43^ | Meta-analysis | 39/ from inception to February 2019 | 8 135 384 | High |
| Maternal smoking during pregnancy | (Global, Europe and USA) | Lange et al. 2018^44^ | Meta-analysis | 295 /January 1985 to February 2016 | NR | High |
| Clinical high-risk state for psychosis | (Global, high-, middle-income countries, Europe and USA) | Salazar de Pablo et al 2021^45^ | Meta-analysis | 35/ from inception to January 2021 | 37 135 | High |
| Childhood sexual abuse | (Global, men, and women) | Stoltenborgh et al. 2011^46^ | Meta-analysis | 217/ January 1980 to January 2008 | 9 911748 | Medium |
|  | (High, middle and, low-income countries) | Kessler et al. 2010^4^ | Population-based | WHO World Mental Health Surveys | 51 945 | 8/10 |
| Maternal paracetamol use during pregnancy | (Global) | Zafeiri et al. 2021^1^ | Systematic review | 16/ NR | 48 613 | Low |
| Insufficient physical activity | (Global, Europe, high, middle and, low-income countries, men and women) | Guthold et al. 2018^39^ | Pooled analysis | 358 surveys across 168 countries between 2001 and 2016 | 1 900 000 | NA |
|  | USA | CDC 2018) ^40^ | CDC report | NA/2008—2018 | NR | NA |
| Childhood adversities | (Global, high, middle and, low-income countries) | Kessler et al. 2010^4^ | Population-based | WHO World Mental Health Surveys | 51 945 | 8/10 |
| Childhood physical abuse | (Global, high, middle and, low-income countries) | Kessler et al. 2010^4^ | Population-based | WHO World Mental Health Surveys | 51 945 | 8/10 |
| Benzodiazepines use | Global and USA | Maust et al. 2019^47^ | Population-based | 2015 and 2016 National Survey on Drug Use and Health data limited to adults ≥18 | 86186 | 7/10 |

NR=Not reported, NA=Not applicable *The OECD Inventory for the Quality of Working Environment covers 7 international surveys that contain information on the Quality of the Working Environment

# eTable 4. Articles excluded after full-text revision, with reasons

| **Author, year** | **Reason for exclusion** |
| --- | --- |
| Angelino, 2019^1^ | Systematic reviews or meta-analyses other than quantitative umbrella reviews, and study protocols |
| Saygın, 2021^2^ | Systematic reviews or meta-analyses other than quantitative umbrella reviews, and study protocols |
| Barbaresko, 2020^3^ | Umbrella reviews employing other classification approaches, such as GRADE |
| Barbui, 2020^4^ | Umbrella reviews addressing outcomes other than the onset of an established mental disorder or biomarkers |
| Belbasis, 2015^5^ | Umbrella reviews addressing outcomes other than the onset of an established mental disorder or biomarkers |
| Belbasis, 2016^6^ | Umbrella reviews addressing outcomes other than the onset of an established mental disorder or biomarkers |
| Belbasis, 2020^7^ | Umbrella reviews addressing outcomes other than the onset of an established mental disorder or biomarkers |
| Bellou, 2016^8^ | Umbrella reviews addressing outcomes other than the onset of an established mental disorder or biomarkers |
| Beynon, 2020^9^ | Systematic reviews or meta-analyses other than quantitative umbrella reviews, and study protocols |
| Bigarella, 2021^10^ | Umbrella reviews addressing outcomes other than the onset of an established mental disorder or biomarkers |
| Bo, 2020^11^ | Umbrella reviews addressing outcomes other than the onset of an established mental disorder or biomarkers |
| Carvalho, 2020^12^ | Umbrella reviews addressing outcomes other than the onset of an established mental disorder or biomarkers |
| Choi, 2021^13^ | Umbrella reviews addressing outcomes other than the onset of an established mental disorder or biomarkers |
| Dadi Abel, 2020^14^ | Umbrella reviews addressing outcomes other than the onset of an established mental disorder or biomarkers |
| De Sio, 2020^15^ | Umbrella reviews addressing outcomes other than the onset of an established mental disorder or biomarkers |
| Demurtas, 2020^16^ | Umbrella reviews addressing outcomes other than the onset of an established mental disorder or biomarkers |
| Dinu, 2018^17^ | Umbrella reviews addressing outcomes other than the onset of an established mental disorder or biomarkers |
| Dragioti, 2019^18^ | Overlapping umbrella review |
| Ehsan, 2019^19^ | Systematic reviews or meta-analyses other than quantitative umbrella reviews, and study protocols |
| Fazel, 2018^20^ | Umbrella reviews addressing outcomes other than the onset of an established mental disorder or biomarkers |
| Farhat, 2020^21^ | Umbrella reviews addressing outcomes other than the onset of an established mental disorder or biomarkers |
| Fullana, 2020^22^ | No class I-II in prospective analysis |
| Fusar-Poli, 2019^23^ | Umbrella reviews addressing outcomes other than the onset of an established mental disorder or biomarkers |
| Galbete, 2018^24^ | Systematic reviews or meta-analyses other than quantitative umbrella reviews, and study protocols |
| Godos, 2020^25^ | Umbrella reviews addressing outcomes other than the onset of an established mental disorder or biomarkers |
| Grosso, 2017^26^ | Umbrella reviews addressing outcomes other than the onset of an established mental disorder or biomarkers |
| Hailes, 2019^27^ | Umbrella reviews employing other classification approaches, such as GRADE |
| Hossain, 2020^28^ | Umbrella reviews addressing outcomes other than the onset of an established mental disorder or biomarkers |
| Hutchens, 2020^29^ | Systematic reviews or meta-analyses other than quantitative umbrella reviews, and study protocols |
| Jayedi, 2020^30^ | Umbrella reviews addressing outcomes other than the onset of an established mental disorder or biomarkers |
| Jayedi, 2020^31^ | Umbrella reviews addressing outcomes other than the onset of an established mental disorder or biomarkers |
| Kelly, 2020^32^ | Systematic reviews or meta-analyses other than quantitative umbrella reviews, and study protocols |
| Lee, 2020^33^ | Umbrella reviews addressing outcomes other than the onset of an established mental disorder or biomarkers |
| Li, 2017^34^ | Umbrella reviews addressing outcomes other than the onset of an established mental disorder or biomarkers |
| Li, 2020^35^ | Umbrella reviews employing other classification approaches, such as GRADE |
| Marventano, 2020^36^ | Umbrella reviews addressing outcomes other than the onset of an established mental disorder or biomarkers |
| McMahon, 2019^37^ | Umbrella reviews addressing outcomes other than the onset of an established mental disorder or biomarkers |
| Mentis, 2021^38^ | Umbrella reviews addressing outcomes other than the onset of an established mental disorder or biomarkers |
| Obaid, 2020^39^ | Umbrella reviews employing other classification approaches, such as GRADE |
| O'Malley, 2021^40^ | Umbrella reviews addressing outcomes other than the onset of an established mental disorder or biomarkers |
| Oussalah, 2020^41^ | Umbrella reviews addressing outcomes other than the onset of an established mental disorder or biomarkers |
| Poole, 2017^42^ | Umbrella reviews addressing outcomes other than the onset of an established mental disorder or biomarkers |
| Posadzki, 2018^43^ | Umbrella reviews addressing outcomes other than the onset of an established mental disorder or biomarkers |
| Solmi, 2020^44^ | No class I-II in prospective analysis |
| Solmi, 2020^45^ | No class I-II in prospective analysis |
| Solmi, 2020^46^ | No class I-II in prospective analysis |
| Solmi, 2020^47^ | Umbrella reviews addressing outcomes other than the onset of an established mental disorder or biomarkers |
| Solmi, 2021^48^ | No class I-II in prospective analysis |
| Solmi, 2021^49^ | No class I-II in prospective analysis |
| Tortella-Feliu, 2020^50^ | No class I-II in prospective analysis |
| van Ijzendoorn, 2020^51^ | Umbrella reviews addressing outcomes other than the onset of an established mental disorder or biomarkers |
| Varchmin, 2021^52^ | Umbrella reviews employing other classification approaches, such as GRADE |
| Veronese, 2018^53^ | Umbrella reviews addressing outcomes other than the onset of an established mental disorder or biomarkers |
| Veronese, 2019^54^ | Umbrella reviews employing other classification approaches, such as GRADE |
| Veronese, 2020^55^ | Umbrella reviews addressing outcomes other than the onset of an established mental disorder or biomarkers |
| Wan, 2019^56^ | Umbrella reviews addressing outcomes other than the onset of an established mental disorder or biomarkers |
| Wolf, 2017^57^ | Umbrella reviews addressing outcomes other than the onset of an established mental disorder or biomarkers |
| Xia, 2020^58^ | Umbrella reviews addressing outcomes other than the onset of an established mental disorder or biomarkers |
| Xu, 2021^59^ | Umbrella reviews addressing outcomes other than the onset of an established mental disorder or biomarkers |
| Yi, 2019^60^ | Umbrella reviews employing other classification approaches, such as GRADE |
| Zhang, 2021^61^ | Umbrella reviews addressing outcomes other than the onset of an established mental disorder or biomarkers |

# eTable 5. Overall characteristics of the umbrella reviews included in the current study

|  | **ICD-10 diagnostic block** | **Number of included meta-analyses** | **Median number of individual studies (IQR) per association** | **Median number of cases (IQR) per association** | **Total number of risk factors tested *** | **Evidence reviewed (years range)** |
| --- | --- | --- | --- | --- | --- | --- |
| Bellou et al. 2017^5^ | Organic, including symptomatic, mental disorders | 43 | 7 (5-13) | 1,139 (590-3,537) | 53 | 2008-2016 |
| Belbasis et al. 2018^6^ | Schizophrenia, schizotypal and delusional disorders | 41 | 7 (5-10) | 384 (254-939) | 41 | 1995-2016 |
| Kim et al. 2019^7^ | Disorders of psychological development | 46 | 8 (2-24) | 3,764(1,000-8,831) | 67 | 2011-2019 |
| Kim et al. 2020^8^ | Behavioural and emotional disorders with onset usually occurring in childhood and adolescence | 35 | 6 (4-8) | 16,850 (1,490–37,086) | 40 | 2012-2020 |
| Kohler et al. 2018^9^ | Mood (affective) disorders | 70 | 7.5 (5-11) | 2,269 (621- 9,090) | 134 | 2003-2017 |
| Radua et al. 2018^10^ | Schizophrenia, schizotypal and delusional disorders | 55 | 5 (3-9) | 424 (226-1,193) | 170 | 1995-2017 |
| Solmi et al. 2021^11^ | Mental and behavioural disorders due to psychoactive substance use | 5 | 10 (7-14) | 634 (366-1,621) | 12 | 2011-2019 |

IQR – interquartile range; * The total includes class I-IV and non-significant risk/protective factors

# eTable 6. Specific PAFs for the most robust risk factors of mental disorders

| **Factor** | **Mental disorder** | **PAF Europe** | **PAF**  **USA** | **PAF high-income**  **countries** | **PAF middle-income countries** | **PAF low-income countries** | **PAF men** | **PAF**  **women** | **PAF** **young people 15–49 years** | **PAF**  **adults≥70 years** |
| --- | --- | --- | --- | --- | --- | --- | --- | --- | --- | --- |
| Childhood adversities | Schizophrenia spectrum disorders | NA | NA | 37.59% | 37.90% | 38.02% | NA | NA | NA | NA |
| Tobacco smoking | Opioid use disorder | 32.46% | 18.37% | 25.77% | 23.86% | 15.26% | 34.45% | 26.99% | NA | NA |
| Job strain | Depressive disorders | NA | NA | NA | NA | NA | NA | NA | NA | NA |
| Insufficient physical activity | Alzheimer's disease | 12.70% | 13.64% | 18.62% | 13.92% | 9.15% | 12.70% | 16.46% | NA | NA |
| Childhood sexual abuse | Depressive disorders | NA | NA | 3.05% | 0.78% | 1.93% | 9.07% | 19.78% | NA | NA |
| Clinical high-risk state for psychosis | Any non-organic psychotic disorder | 13.00% | 21.51% | 13.00% | 9.06% | NA | NA | NA | NA | NA |
| Maternal paracetamol use during pregnancy* | ADHD | NA | NA | NA | NA | NA | NA | NA | NA | NA |
| Three metabolic risk factors | Depressive disorders | NA | NA | NA | NA | NA | 4.25% | 3.74% | NA | NA |
| Cannabis use | Schizophrenia spectrum disorders | 13.26% | 19.98% | NA | NA | NA | NA | NA | NA | NA |
| Maternal pre-pregnancy obesity | ADHD | 5.41% | 9.96% | NA | NA | NA | NA | 9.30% | NA | NA |
| Type 2 diabetes mellitus | Vascular dementia | 9.01% | 9.28% | 10.25% | 6.90% | 3.14% | 6.93% | 6.53% | 3.76% | 22.84% |
| Childhood physical abuse | Depressive disorders | NA | NA | 4.47% | 8.71% | 7.37% | NA | NA | NA | NA |
| Maternal overweight pre/during pregnancy | Autism spectrum disorder | 5.54% | 7.46% | NA | NA | NA | NA | 6.47% | NA | NA |
| Maternal overweight pre/during pregnancy | ADHD | 5.15% | 6.95% | NA | NA | NA | NA | 6.02% | NA | NA |
| Benzodiazepines use* | Any dementia | NA | 5.84% | NA | NA | NA | NA | NA | NA | NA |
| Four or five metabolic risk factors | Depressive disorders | NA | NA | NA | NA | NA | 7.30% | 8.31% | NA | 35.14% |
| Depression in elderhood | Any dementia | 4.31% | 3.46% | 3.28% | 4.38% | 5.98% | 3.55% | 4.87% | NA | 4.30% |
| Type 2 diabetes mellitus | Any dementia | 4.45% | 4.59% | 5.10% | 3.37% | 1.50% | 3.39% | 3.18% | 1.80% | 12.22% |
| Depression in elderhood | Alzheimer's disease | 3.35% | 2.69% | 2.54% | 3.41% | 4.67% | 2.76% | 3.80% | 3.53% | 4.44% |
| Depression | Any dementia | 3.52% | 3.23% | 3.36% | 2.89% | 2.70% | 2.35% | 3.65% | 1.63% | 11.19% |
| Type 2 diabetes mellitus | Alzheimer's disease | 4.04% | 4.17% | 4.64% | 3.06% | 1.36% | 3.07% | 2.89% | NA | NA |
| Obesity | Depressive disorders | 5.28% | 7.60% | 2.54% | 0.95% | 0.42% | 3.21% | 4.62% | 2.98% | 3.75% |
| Depression | Alzheimer's disease | 2.97% | 2.73% | 2.83% | 2.44% | 2.27% | 1.98% | 3.09% | NA | NA |
| Maternal SSRI use during pregnancy* | Autism spectrum disorder | 1.06% | 3.44% | NA | NA | NA | NA | 1.92% | NA | NA |
| Maternal smoking during pregnancy | ADHD | 4.52% | 3.34% | NA | NA | NA | NA | 15,76% | NA | NA |

ADHD – attention deficit/hyperactivity disorder, NA- not applicable, SSRI– selective serotonin reuptake inhibitors, * documented or likely confounding by indication.

# eFigure 1. Forest plot for the reversed factor of high physical activity to insufficient physical activity (Beckett et al. 2015)^20^

eFigure 2. Meta-analysis of the proportion of four or five metabolic risk factors (Jokela et al. 2014)^3^

eFigure 3. Meta-analysis of the proportion of three metabolic risk factors (Jokela et al. 2014)^3^

# eFigure 4. Meta-analysis of the proportion of maternal paracetamol use during pregnancy (Zafeiri et al. 2021)^1^

# eFigure 5. The meta-analytic Generalized Impact Fraction for insufficient physical activity


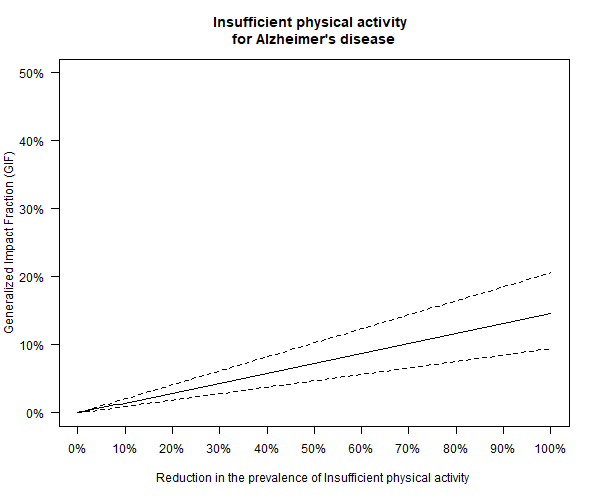


# eFigure 6. The meta-analytic Generalized Impact Fraction for tobacco smoking


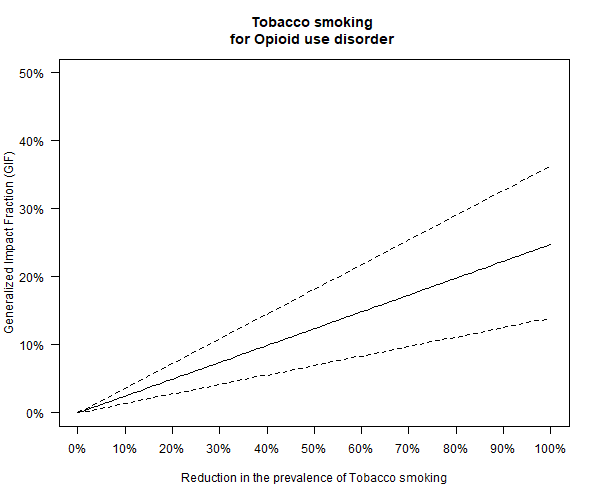


# eFigure 7. The meta-analytic Generalized Impact Fraction for job strain


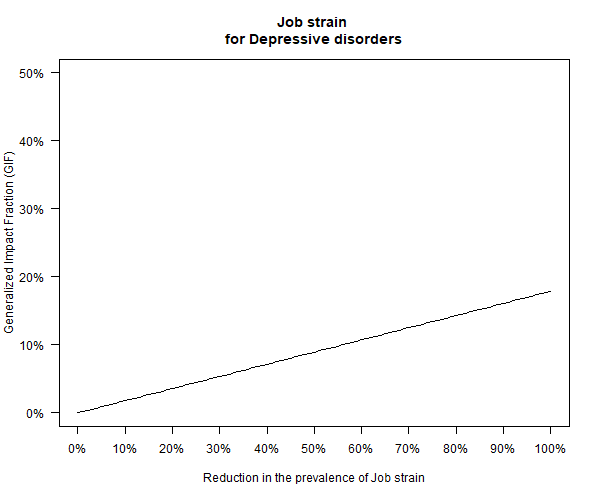


Notes: CIs not predictable

# eFigure 8. The meta-analytic Generalized Impact Fraction for sexual abuse in childhood (a) and three metabolic risk factors (b)


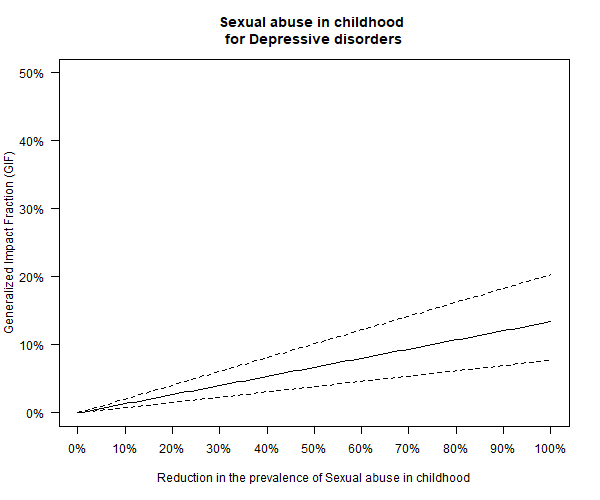

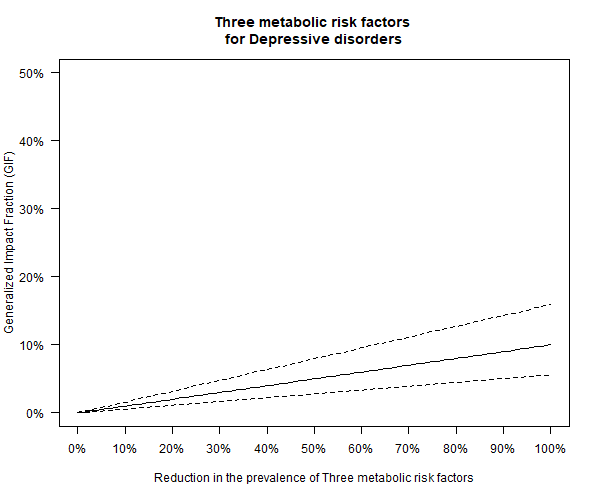


a

b

# eFigure 9. The meta-analytic Generalized Impact Fraction for maternal pre-pregnancy obesity


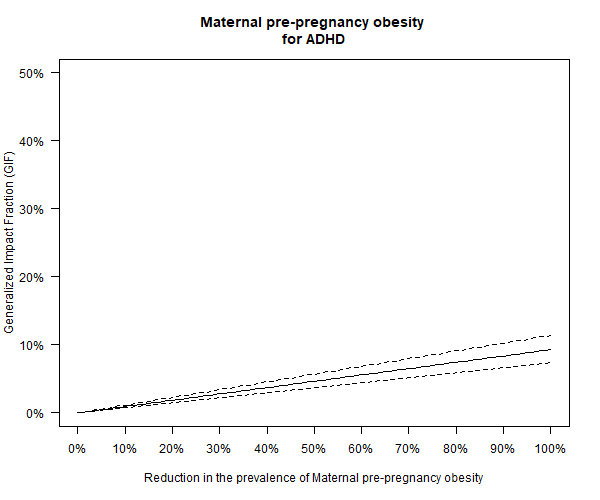


# eFigure 10. The meta-analytic country-level PAF for tobacco smoking and opioid use disorder

# eFigure 11. The meta-analytic country-level PAF for job strain and depressive disorders

# **eReferences**

1. Zafeiri A, Mitchell RT, Hay DC, Fowler PA. Over-the-counter analgesics during pregnancy: a comprehensive review of global prevalence and offspring safety. *Hum Reprod Update* 2021; **27**(1): 67-95.

2. Nyaga VN, Arbyn M, Aerts M. Metaprop: a Stata command to perform meta-analysis of binomial data. *Arch Public Health* 2014; **72**(1): 39.

3. Jokela M, Hamer M, Singh-Manoux A, Batty GD, Kivimaki M. Association of metabolically healthy obesity with depressive symptoms: pooled analysis of eight studies. *Mol Psychiatry* 2014; **19**(8): 910-4.

4. Kessler RC, McLaughlin KA, Green JG, et al. Childhood adversities and adult psychopathology in the WHO World Mental Health Surveys. *Br J Psychiatry* 2010; **197**(5): 378-85.

5. Bellou V, Belbasis L, Tzoulaki I, Middleton LT, Ioannidis JPA, Evangelou E. Systematic evaluation of the associations between environmental risk factors and dementia: An umbrella review of systematic reviews and meta-analyses. *Alzheimers Dement* 2017; **13**(4): 406-18.

6. Belbasis L, Kohler CA, Stefanis N, et al. Risk factors and peripheral biomarkers for schizophrenia spectrum disorders: an umbrella review of meta-analyses. *Acta Psychiatr Scand* 2018; **137**(2): 88-97.

7. Kim JY, Son MJ, Son CY, et al. Environmental risk factors and biomarkers for autism spectrum disorder: an umbrella review of the evidence. *Lancet Psychiatry* 2019; **6**(7): 590-600.

8. Kim JH, Kim JY, Lee J, et al. Environmental risk factors, protective factors, and peripheral biomarkers for ADHD: an umbrella review. *Lancet Psychiatry* 2020; **7**(11): 955-70.

9. Kohler CA, Evangelou E, Stubbs B, et al. Mapping risk factors for depression across the lifespan: An umbrella review of evidence from meta-analyses and Mendelian randomization studies. *J Psychiatr Res* 2018; **103**: 189-207.

10. Radua J, Ramella-Cravaro V, Ioannidis JPA, et al. What causes psychosis? An umbrella review of risk and protective factors. *World Psychiatry* 2018; **17**(1): 49-66.

11. Solmi M, Dragioti E, Croatto G, et al. Risk and protective factors for cannabis, cocaine, and opioid use disorders: An umbrella review of meta-analyses of observational studies. *Neurosci Biobehav Rev* 2021; **126**: 243-51.

12. Marconi A, Di Forti M, Lewis CM, Murray RM, Vassos E. Meta-analysis of the Association Between the Level of Cannabis Use and Risk of Psychosis. *Schizophr Bull* 2016; **42**(5): 1262-9.

13. da Silva J, Goncalves-Pereira M, Xavier M, Mukaetova-Ladinska EB. Affective disorders and risk of developing dementia: systematic review. *Br J Psychiatry* 2013; **202**(3): 177-86.

14. Diniz BS, Butters MA, Albert SM, Dew MA, Reynolds CF, 3rd. Late-life depression and risk of vascular dementia and Alzheimer's disease: systematic review and meta-analysis of community-based cohort studies. *Br J Psychiatry* 2013; **202**(5): 329-35.

15. Rajabi A, Dehghani M, Shojaei A, Farjam M, Motevalian SA. Association between tobacco smoking and opioid use: A meta-analysis. *Addict Behav* 2019; **92**: 225-35.

16. Gudala K, Bansal D, Schifano F, Bhansali A. Diabetes mellitus and risk of dementia: A meta-analysis of prospective observational studies. *J Diabetes Investig* 2013; **4**(6): 640-50.

17. Madsen IEH, Nyberg ST, Magnusson Hanson LL, et al. Job strain as a risk factor for clinical depression: systematic review and meta-analysis with additional individual participant data. *Psychol Med* 2017; **47**(8): 1342-56.

18. Atlantis E, Sullivan T. Bidirectional association between depression and sexual dysfunction: a systematic review and meta-analysis. *J Sex Med* 2012; **9**(6): 1497-507.

19. Bao YP, Han Y, Ma J, et al. Cooccurrence and bidirectional prediction of sleep disturbances and depression in older adults: Meta-analysis and systematic review. *Neurosci Biobehav Rev* 2017; **75**: 257-73.

20. Beckett MW, Ardern CI, Rotondi MA. A meta-analysis of prospective studies on the role of physical activity and the prevention of Alzheimer's disease in older adults. *BMC Geriatr* 2015; **15**: 9.

21. Kuiper JS, Zuidersma M, Oude Voshaar RC, et al. Social relationships and risk of dementia: A systematic review and meta-analysis of longitudinal cohort studies. *Ageing Res Rev* 2015; **22**: 39-57.

22. Wang Y, Tang S, Xu S, Weng S, Liu Z. Maternal Body Mass Index and Risk of Autism Spectrum Disorders in Offspring: A Meta-analysis. *Sci Rep* 2016; **6**: 34248.

23. Jenabi E, Bashirian S, Khazaei S, Basiri Z. The maternal prepregnancy body mass index and the risk of attention deficit hyperactivity disorder among children and adolescents: a systematic review and meta-analysis. *Korean J Pediatr* 2019; **62**(10): 374-9.

24. Andalib S, Emamhadi MR, Yousefzadeh-Chabok S, et al. Maternal SSRI exposure increases the risk of autistic offspring: A meta-analysis and systematic review. *Eur Psychiatry* 2017; **45**: 161-6.

25. Huang L, Wang Y, Zhang L, et al. Maternal Smoking and Attention Deficit/Hyperactivity Disorder in Offspring: A Meta-analysis. *Pediatrics* 2018; **141**(1).

26. Fusar-Poli P, Cappucciati M, Rutigliano G, et al. At risk or not at risk? A meta-analysis of the prognostic accuracy of psychometric interviews for psychosis prediction. *World Psychiatry* 2015; **14**(3): 322-32.

27. Mandelli L, Petrelli C, Serretti A. The role of specific early trauma in adult depression: A meta-analysis of published literature. Childhood trauma and adult depression. *Eur Psychiatry* 2015; **30**(6): 665-80.

28. Gou X, Wang Y, Tang Y, et al. Association of maternal prenatal acetaminophen use with the risk of attention deficit/hyperactivity disorder in offspring: A meta-analysis. *Aust N Z J Psychiatry* 2019; **53**(3): 195-206.

29. Varese F, Smeets F, Drukker M, et al. Childhood adversities increase the risk of psychosis: a meta-analysis of patient-control, prospective- and cross-sectional cohort studies. *Schizophr Bull* 2012; **38**(4): 661-71.

30. Zhong G, Wang Y, Zhang Y, Zhao Y. Association between Benzodiazepine Use and Dementia: A Meta-Analysis. *PLoS One* 2015; **10**(5): e0127836.

31. <https://www.unodc.org/wdr2017/field/Booklet_1_EXSUM.pdf>.

32. <https://dataunodc.un.org/data/drugs/Prevalence-general>.

33. <http://ghdx.healthdata.org/gbd-results-tool?params=gbd-api-2019-permalink/99e84a6a49f07cab05207e95551c3156>.

34. <http://ghdx.healthdata.org/record/ihme-data/gbd-2015-obesity-and-overweight-prevalence-1980-2015>.

35. WHO global report on trends in prevalence of tobacco smoking 2000-2025, third Edition, Geveva; World Health Organization, (2019).

36. Jafari A, Rajabi A, Gholian-Aval M, Peyman N, Mahdizadeh M, Tehrani H. National, regional, and global prevalence of cigarette smoking among women/females in the general population: a systematic review and meta-analysis. *Environ Health Prev Med* 2021; **26**(1): 5.

37. Cornelius ME, Wang TW, Jamal A, Loretan CG, Neff LJ. Tobacco Product Use Among Adults - United States, 2019. *MMWR Morb Mortal Wkly Rep* 2020; **69**(46): 1736-42.

38. <http://stats.oecd.org/Index.aspx?DataSetCode=JOBQ>.

39. Guthold R, Stevens GA, Riley LM, Bull FC. Worldwide trends in insufficient physical activity from 2001 to 2016: a pooled analysis of 358 population-based surveys with 1.9 million participants. *Lancet Glob Health* 2018; **6**(10): e1077-e86.

40. <https://www.cdc.gov/physicalactivity/downloads/trends-in-the-prevalence-of-physical-activity-508.pdf>.

41. Kuk JL, Ardern CI. Age and sex differences in the clustering of metabolic syndrome factors: association with mortality risk. *Diabetes Care* 2010; **33**(11): 2457-61.

42. Martinez-Hortelano JA, Cavero-Redondo I, Alvarez-Bueno C, Garrido-Miguel M, Soriano-Cano A, Martinez-Vizcaino V. Monitoring gestational weight gain and prepregnancy BMI using the 2009 IOM guidelines in the global population: a systematic review and meta-analysis. *BMC Pregnancy Childbirth* 2020; **20**(1): 649.

43. Molenaar NM, Bais B, Lambregtse-van den Berg MP, et al. The international prevalence of antidepressant use before, during, and after pregnancy: A systematic review and meta-analysis of timing, type of prescriptions and geographical variability. *J Affect Disord* 2020; **264**: 82-9.

44. Lange S, Probst C, Rehm J, Popova S. National, regional, and global prevalence of smoking during pregnancy in the general population: a systematic review and meta-analysis. *Lancet Glob Health* 2018; **6**(7): e769-e76.

45. Salazar de Pablo G, Woods WS, Drymonitou G, de Diego H; Fusar-Poli P. Prevalence of Individuals at Clinical High-risk of Psychosis in the General Population and Clinical Samples: Systematic Review and Meta-analysis. *Under review* 2021.

46. Stoltenborgh M, van Ijzendoorn MH, Euser EM, Bakermans-Kranenburg MJ. A global perspective on child sexual abuse: meta-analysis of prevalence around the world. *Child Maltreat* 2011; **16**(2): 79-101.

47. Maust DT, Lin LA, Blow FC. Benzodiazepine Use and Misuse Among Adults in the United States. *Psychiatr Serv* 2019; **70**(2): 97-106.

# References of excluded umbrella reviews with reasons

1. Angelino D, Godos J, Ghelfi F, et al. Fruit and vegetable consumption and health outcomes: an umbrella review of observational studies. Int J Food Sci Nutr. 2019;70(6):652-667. doi:10.1080/09637486.2019.1571021
2. Avşar TS, McLeod H, Jackson L. Health outcomes of smoking during pregnancy and the postpartum period: an umbrella review. BMC Pregnancy Childbirth. 2021;21(1):254. Published 2021 Mar 26. doi:10.1186/s12884-021-03729-1
3. Barbaresko J, Lellmann AW, Schmidt A, et al. Dietary Factors and Neurodegenerative Disorders: An Umbrella Review of Meta-Analyses of Prospective Studies. Adv Nutr. 2020;11(5):1161-1173. doi:10.1093/advances/nmaa053
4. Barbui C, Purgato M, Abdulmalik J, et al. Efficacy of psychosocial interventions for mental health outcomes in low-income and middle-income countries: an umbrella review. Lancet Psychiatry. 2020;7(2):162-172. doi:10.1016/S2215-0366(19)30511-5
5. Belbasis L, Bellou V, Evangelou E, Ioannidis JP, Tzoulaki I. Environmental risk factors and multiple sclerosis: an umbrella review of systematic reviews and meta-analyses. Lancet Neurol. 2015;14(3):263-273. doi:10.1016/S1474-4422(14)70267-4
6. Belbasis L, Bellou V, Evangelou E. Environmental Risk Factors and Amyotrophic Lateral Sclerosis: An Umbrella Review and Critical Assessment of Current Evidence from Systematic Reviews and Meta-Analyses of Observational Studies. Neuroepidemiology. 2016;46(2):96-105. doi:10.1159/000443146
7. Belbasis L, Bellou V, Evangelou E, Tzoulaki I. Environmental factors and risk of multiple sclerosis: Findings from meta-analyses and Mendelian randomization studies. Mult Scler. 2020;26(4):397-404. doi:10.1177/1352458519872664
8. Bellou V, Belbasis L, Tzoulaki I, Evangelou E, Ioannidis JP. Environmental risk factors and Parkinson's disease: An umbrella review of meta-analyses. Parkinsonism Relat Disord. 2016;23:1-9. doi:10.1016/j.parkreldis.2015.12.008
9. Beynon C, Pearce-Smith N, Clark R. Risk factors for gambling and problem gambling: a protocol for a rapid umbrella review of systematic reviews and meta-analyses. Syst Rev. 2020;9(1):198. Published 2020 Aug 27. doi:10.1186/s13643-020-01455-x
10. Bigarella LG, Ballotin VR, Mazurkiewicz LF, et al. Exercise for depression and depressive symptoms in older adults: an umbrella review of systematic reviews and Meta-analyses [published online ahead of print, 2021 Jul 30]. Aging Ment Health. 2021;1-11. doi:10.1080/13607863.2021.1951660
11. Bo Y, Zhu Y, Tao Y, et al. Association Between Folate and Health Outcomes: An Umbrella Review of Meta-Analyses. Front Public Health. 2020;8:550753. Published 2020 Dec 15. doi:10.3389/fpubh.2020.550753
12. Carvalho AF, Solmi M, Sanches M, et al. Evidence-based umbrella review of 162 peripheral biomarkers for major mental disorders. Transl Psychiatry. 2020;10(1):152. Published 2020 May 18. doi:10.1038/s41398-020-0835-5
13. Choi J, Price J, Ryder S, Siskind D, Solmi M, Kisely S. Prevalence of dental disorders among people with mental illness: An umbrella review [published online ahead of print, 2021 Aug 30]. Aust N Z J Psychiatry. 2021;48674211042239. doi:10.1177/00048674211042239
14. Dadi AF, Miller ER, Bisetegn TA, Mwanri L. Global burden of antenatal depression and its association with adverse birth outcomes: an umbrella review. BMC Public Health. 2020;20(1):173. Published 2020 Feb 4. doi:10.1186/s12889-020-8293-9
15. De Sio S, Buomprisco G, Perri R, et al. Work-related stress risk and preventive measures of mental disorders in the medical environment: an umbrella review. Eur Rev Med Pharmacol Sci. 2020;24(2):821-830. doi:10.26355/eurrev_202001_20065
16. Demurtas J, Schoene D, Torbahn G, et al. Physical Activity and Exercise in Mild Cognitive Impairment and Dementia: An Umbrella Review of Intervention and Observational Studies. J Am Med Dir Assoc. 2020;21(10):1415-1422.e6. doi:10.1016/j.jamda.2020.08.031
17. Dinu M, Pagliai G, Casini A, Sofi F. Mediterranean diet and multiple health outcomes: an umbrella review of meta-analyses of observational studies and randomised trials. Eur J Clin Nutr. 2018;72(1):30-43. doi:10.1038/ejcn.2017.58
18. Dragioti E, Solmi M, Favaro A, et al. Association of Antidepressant Use With Adverse Health Outcomes: A Systematic Umbrella Review. JAMA Psychiatry. 2019;76(12):1241-1255. doi:10.1001/jamapsychiatry.2019.2859
19. Ehsan A, Klaas HS, Bastianen A, Spini D. Social capital and health: A systematic review of systematic reviews. SSM Popul Health. 2019;8:100425. Published 2019 Jun 7. doi:10.1016/j.ssmph.2019.100425
20. Fazel S, Smith EN, Chang Z, Geddes JR. Risk factors for interpersonal violence: an umbrella review of meta-analyses. Br J Psychiatry. 2018;213(4):609-614. doi:10.1192/bjp.2018.145
21. Farhat LC, Carvalho AF, Solmi M, Brunoni AR. Evidence-based Umbrella Review of Cognitive Effects of Prefrontal tDCS [published online ahead of print, 2020 Jun 24]. Soc Cogn Affect Neurosci. 2020;nsaa084. doi:10.1093/scan/nsaa084
22. Fullana MA, Tortella-Feliu M, Fernández de la Cruz L, et al. Risk and protective factors for anxiety and obsessive-compulsive disorders: an umbrella review of systematic reviews and meta-analyses. Psychol Med. 2020;50(8):1300-1315. doi:10.1017/S0033291719001247
23. Fusar-Poli P, Davies C, Solmi M, et al. Preventive Treatments for Psychosis: Umbrella Review (Just the Evidence). Front Psychiatry. 2019;10:764. Published 2019 Dec 11. doi:10.3389/fpsyt.2019.00764
24. Galbete C, Schwingshackl L, Schwedhelm C, Boeing H, Schulze MB. Evaluating Mediterranean diet and risk of chronic disease in cohort studies: an umbrella review of meta-analyses. Eur J Epidemiol. 2018;33(10):909-931. doi:10.1007/s10654-018-0427-3
25. Godos J, Tieri M, Ghelfi F, et al. Dairy foods and health: an umbrella review of observational studies. Int J Food Sci Nutr. 2020;71(2):138-151. doi:10.1080/09637486.2019.1625035
26. Grosso G, Godos J, Galvano F, Giovannucci EL. Coffee, Caffeine, and Health Outcomes: An Umbrella Review. Annu Rev Nutr. 2017;37:131-156. doi:10.1146/annurev-nutr-071816-064941
27. Hailes HP, Yu R, Danese A, Fazel S. Long-term outcomes of childhood sexual abuse: an umbrella review. Lancet Psychiatry. 2019;6(10):830-839. doi:10.1016/S2215-0366(19)30286-X
28. Hossain MM, Khan N, Sultana A, et al. Prevalence of comorbid psychiatric disorders among people with autism spectrum disorder: An umbrella review of systematic reviews and meta-analyses. Psychiatry Res. 2020;287:112922. doi:10.1016/j.psychres.2020.112922
29. Hutchens BF, Kearney J. Risk Factors for Postpartum Depression: An Umbrella Review. J Midwifery Womens Health. 2020;65(1):96-108. doi:10.1111/jmwh.13067
30. Jayedi A, Shab-Bidar S. Fish Consumption and the Risk of Chronic Disease: An Umbrella Review of Meta-Analyses of Prospective Cohort Studies. Adv Nutr. 2020;11(5):1123-1133. doi:10.1093/advances/nmaa029
31. Jayedi A, Soltani S, Abdolshahi A, Shab-Bidar S. Healthy and unhealthy dietary patterns and the risk of chronic disease: an umbrella review of meta-analyses of prospective cohort studies. Br J Nutr. 2020;124(11):1133-1144. doi:10.1017/S0007114520002330
32. Kelly MM, Griffith PB. Umbrella Review of School Age Health Outcomes of Preterm Birth Survivors. J Pediatr Health Care. 2020;34(5):e59-e76. doi:10.1016/j.pedhc.2020.05.007
33. Lee J, Son MJ, Son CY, et al. Genetic Variation and Autism: A Field Synopsis and Systematic Meta-Analysis. Brain Sci. 2020;10(10):692. Published 2020 Sep 30. doi:10.3390/brainsci10100692
34. Li X, Meng X, Timofeeva M, et al. Serum uric acid levels and multiple health outcomes: umbrella review of evidence from observational studies, randomised controlled trials, and Mendelian randomisation studies [published correction appears in BMJ. 2017 Aug 8;358:j3799]. BMJ. 2017;357:j2376.
35. Li N, Wu X, Zhuang W, et al. “Fish consumption and multiple health outcomes: Umbrella review.” Trends in Food Science and Technology 99 (2020): 273-283.
36. Marventano S, Godos J, Tieri M, et al. Egg consumption and human health: an umbrella review of observational studies. Int J Food Sci Nutr. 2020;71(3):325-331. doi:10.1080/09637486.2019.1648388
37. McMahon N, Thomson K, Kaner E, Bambra C. Effects of prevention and harm reduction interventions on gambling behaviours and gambling related harm: An umbrella review. Addict Behav. 2019;90:380-388. doi:10.1016/j.addbeh.2018.11.048
38. Mentis AA, Dardiotis E, Efthymiou V, Chrousos GP. Non-genetic risk and protective factors and biomarkers for neurological disorders: a meta-umbrella systematic review of umbrella reviews. BMC Med. 2021;19(1):6. Published 2021 Jan 13. doi:10.1186/s12916-020-01873-7
39. Obaid M, Douiri A, Flach C, Prasad V, Marshall I. Can we prevent poststroke cognitive impairment? An umbrella review of risk factors and treatments. BMJ Open. 2020;10(9):e037982. Published 2020 Sep 9. doi:10.1136/bmjopen-2020-037982
40. O'Malley N, Clifford AM, Conneely M, Casey B, Coote S. Effectiveness of interventions to prevent falls for people with multiple sclerosis, Parkinson's disease and stroke: an umbrella review. BMC Neurol. 2021;21(1):378. Published 2021 Sep 29. doi:10.1186/s12883-021-02402-6
41. Oussalah A, Levy J, Berthezène C, Alpers DH, Guéant JL. Health outcomes associated with vegetarian diets: An umbrella review of systematic reviews and meta-analyses. Clin Nutr. 2020;39(11):3283-3307. doi:10.1016/j.clnu.2020.02.037
42. Poole R, Kennedy OJ, Roderick P, Fallowfield JA, Hayes PC, Parkes J. Coffee consumption and health: umbrella review of meta-analyses of multiple health outcomes. BMJ. 2017;359:j5024. Published 2017 Nov 22. doi:10.1136/bmj.j5024
43. Posadzki PP, Bajpai R, Kyaw BM, et al. Melatonin and health: an umbrella review of health outcomes and biological mechanisms of action. BMC Med. 2018;16(1):18. Published 2018 Feb 5. doi:10.1186/s12916-017-1000-8
44. Solmi M, Radua J, Stubbs B, et al. Risk factors for eating disorders: an umbrella review of published meta-analyses. Braz J Psychiatry. 2021;43(3):314-323. doi:10.1590/1516-4446-2020-1099
45. Solmi M, Dragioti E, Arango C, et al. Risk and protective factors for mental disorders with onset in childhood/adolescence: An umbrella review of published meta-analyses of observational longitudinal studies. Neurosci Biobehav Rev. 2021;120:565-573. doi:10.1016/j.neubiorev.2020.09.002
46. Solmi M, Civardi S, Corti R, et al. Risk and protective factors for alcohol and tobacco related disorders: An umbrella review of observational studies. Neurosci Biobehav Rev. 2021;121:20-28. doi:10.1016/j.neubiorev.2020.11.010
47. Solmi M, Veronese N, Galvano D, et al. Factors Associated With Loneliness: An Umbrella Review Of Observational Studies. J Affect Disord. 2020;271:131-138. doi:10.1016/j.jad.2020.03.075
48. Solmi M, Dragioti E, Croatto G, et al. Risk and Protective Factors for Personality Disorders: An Umbrella Review of Published Meta-Analyses of Case-Control and Cohort Studies. Front Psychiatry. 2021;12:679379. Published 2021 Sep 6. doi:10.3389/fpsyt.2021.679379
49. Solmi M, Dragioti E, Croatto G, et al. Risk and protective factors for cannabis, cocaine, and opioid use disorders: An umbrella review of meta-analyses of observational studies. Neurosci Biobehav Rev. 2021;126:243-251. doi:10.1016/j.neubiorev.2021.03.014
50. Tortella-Feliu M, Fullana MA, Pérez-Vigil A, et al. Risk factors for posttraumatic stress disorder: An umbrella review of systematic reviews and meta-analyses. Neurosci Biobehav Rev. 2019;107:154-165. doi:10.1016/j.neubiorev.2019.09.013
51. van IJzendoorn MH, Bakermans-Kranenburg MJ, Coughlan B, Reijman S. Annual Research Review: Umbrella synthesis of meta-analyses on child maltreatment antecedents and interventions: differential susceptibility perspective on risk and resilience. J Child Psychol Psychiatry. 2020;61(3):272-290. doi:10.1111/jcpp.13147
52. Varchmin L, Montag C, Treusch Y, Kaminski J, Heinz A. Traumatic Events, Social Adversity and Discrimination as Risk Factors for Psychosis - An Umbrella Review. Front Psychiatry. 2021;12:665957. Published 2021 Oct 22. doi:10.3389/fpsyt.2021.665957
53. Veronese N, Solmi M, Caruso MG, et al. Dietary fiber and health outcomes: an umbrella review of systematic reviews and meta-analyses. Am J Clin Nutr. 2018;107(3):436-444. doi:10.1093/ajcn/nqx082
54. Veronese N, Demurtas J, Celotto S, et al. Is chocolate consumption associated with health outcomes? An umbrella review of systematic reviews and meta-analyses. Clin Nutr. 2019;38(3):1101-1108. doi:10.1016/j.clnu.2018.05.019
55. Veronese N, Galvano D, D'Antiga F, et al. Interventions for reducing loneliness: An umbrella review of intervention studies. Health Soc Care Community. 2021;29(5):e89-e96. doi:10.1111/hsc.13248
56. Wan Q, Li N, Du L, et al. Allium vegetable consumption and health: An umbrella review of meta-analyses of multiple health outcomes. Food Sci Nutr. 2019;7(8):2451-2470. Published 2019 Jul 10. doi:10.1002/fsn3.1117
57. Wolf A, Whiting D, Fazel S. Violence prevention in psychiatry: an umbrella review of interventions in general and forensic psychiatry. J Forens Psychiatry Psychol. 2017;28(5):659-673. doi:10.1080/14789949.2017.1284886
58. Xia L, Zhao R, Wan Q, et al. Sarcopenia and adverse health-related outcomes: An umbrella review of meta-analyses of observational studies. Cancer Med. 2020;9(21):7964-7978. doi:10.1002/cam4.3428
59. Xu Y, Zeng L, Zou K, et al. Role of dietary factors in the prevention and treatment for depression: an umbrella review of meta-analyses of prospective studies. Transl Psychiatry. 2021;11(1):478. Published 2021 Sep 16. doi:10.1038/s41398-021-01590-6
60. Keller A, Wallace TC. Tea intake and cardiovascular disease: an umbrella review. Ann Med. 2021;53(1):929-944. doi:10.1080/07853890.2021.1933164
61. Zhang X, Chen X, Xu Y, et al. Milk consumption and multiple health outcomes: umbrella review of systematic reviews and meta-analyses in humans. Nutr Metab (Lond). 2021;18(1):7. Published 2021 Jan 7. doi:10.1186/s12986-020-00527-y
